# Disruption to schizophrenia-associated gene *Fez1* in the hippocampus of HDAC11 knockout mice

**DOI:** 10.1038/s41598-017-11630-1

**Published:** 2017-09-19

**Authors:** Dale T. Bryant, Christian Landles, Aikaterini S. Papadopoulou, Agnesska C. Benjamin, Joshua K. Duckworth, Thomas Rosahl, Caroline L. Benn, Gillian P. Bates

**Affiliations:** 1 UCL Huntington’s Disease Centre, Sobell Department of Motor Neuroscience, UCL Institute of Neurology, University College London, London, United Kingdom; 2 Neusentis, Pfizer Ltd, The Portway, Granta Park, Abington, Cambridge United Kingdom; 3 Merck & Co., Inc, Kenilworth, NJ USA

## Abstract

Histone Deacetylase 11 (HDAC11) is highly expressed in the central nervous system where it has been reported to have roles in neural differentiation. In contrast with previous studies showing nuclear and cytoplasmic localisation, we observed synaptic enrichment of HDAC11. Knockout mouse models for HDACs 1–9 have been important for guiding the development of isoform specific HDAC inhibitors as effective therapeutics. Given the close relationship between HDAC11 and neural cells *in vitro*, we examined neural tissue in a previously uncharacterised *Hdac11* knockout mouse (*Hdac11* ^KO/KO^). Loss of HDAC11 had no obvious impact on brain morphology and neural stem/precursor cells isolated from *Hdac11* ^KO/KO^ mice had comparable proliferation and differentiation characteristics. However, in differentiating neural cells we observed decreased expression of schizophrenia-associated gene *Fez1* (fasciculation and elongation protein zeta 1), a gene previously reported to be regulated by HDAC11 activity. FEZ1 has been associated with the dendritic growth of neurons and risk of schizophrenia via its interaction with DISC1 (disrupted in schizophrenia 1). Examination of cortical, cerebellar and hippocampal tissue reveal decreased *Fez1* expression specifically in the hippocampus of adult mice. The results of this study demonstrate that loss of HDAC11 has age dependent and brain-region specific consequences.

## Introduction

Histone deacetylase 11 (HDAC11) is the most recently identified member of the HDAC family^1^, with homologues identified in most species examined^2,3^. HDAC11 has a single lysine deacetylase domain surrounded by a short N- and C-terminus^1^. This lysine deacetylase domain is shared by all zinc-dependant HDACs (HDAC1-11) and is predicted to catalyse the removal of acetyl groups from acetylated lysine residues. HDAC11 is similar to both class I (HDAC1, -2, -3, and -8) and class II (HDAC4, -5, -6, -7, -9, and -10) zinc-dependent HDACs^3^. As there is little justification for assigning HDAC11 specifically to class I or class II HDACs, it is considered exclusively as the first and only ‘class IV’ HDAC identified to date^4^. The catalytic activity of HDAC11 is inhibited by HDAC inhibitors such as Mocetinostat, Vorinostat, Panobinostat and Quinostat at nanomolar concentrations^5,6^. Recently there have been reports of an inhibitor predicted to specifically target HDAC11^7^.

The expression of *HDAC11* is specific to certain tissues, including the central nervous system^1^. Compared to the other HDACs, *Hdac11* mRNA is particularly abundant in the rat brain with a peculiar pattern of expression in the hippocampus where it is most concentrated in the CA1 (Cornu Ammonis 1) region^8^. HDAC11 appears to be closely related to cell proliferation/differentiation as its expression is mutually exclusive with proliferative marker Ki-67 and its expression increases as neural cells differentiate *in vitro* ^9–11^. Overexpression of HDAC11 in mouse fibroblasts inhibits their proliferation^12^. The *Hdac11* gene and its chromosomal region are associated with variability in regional brain volume of mice^13,14^ and humans^15^. Additionally, multiple reports suggest associations between HDAC11 and malignant disease^16–21^. Two studies have implicated HDAC11 as a negative regulator of cell cycle component DNA replication factor Cdt1 (chromatin licensing and DNA replication factor 1)^22,23^. Whereas other studies have shown a role for HDAC11 in the response of antigen presenting cells^24,25^ and the differentiation of neural cells^10,26^. Watanabe *et al*., demonstrated that knockdown of HDAC11 inhibits dendrite growth of neurons generated in the adult mouse hippocampus^26^. This appeared to result from a regulatory role upstream of FEZ1 (fasciculation and elongation protein zeta 1), as they show that HDAC11 deacetylates BUBR1 (Budding uninhibited by benzimidazole 1-related protein kinase), relieving its inhibition of CDC20/APC (cell division cycle 20/anaphase-promoting complex), allowing that to ubiquitinate FEZ1 and therefore its degradation.

Knockout mouse models for HDACs 1–9 have demonstrated the consequences resulting from complete loss of each individual HDAC^27,28^. As previous studies report a role for HDAC11 in cell proliferation and neural differentiation, the aim of this study was to examine the nervous system in a previously uncharacterised *Hdac11* knockout mouse. *Hdac11* ^KO/KO^ mice were born at Mendelian ratios and brain morphology appeared normal suggesting that HDAC11 does not have a critical role during development. Accordingly, *in vitro* studies on neural cell lines generated from *Hdac11* ^KO/KO^ mice showed normal proliferation patterns. However, we found that the expression of *Fez1* was decreased in both proliferating and differentiating neural cell cultures. We further observed specific decrease in *Fez1* gene expression in the hippocampi of adult *Hdac11* ^KO/KO^ mice. This suggests that HDAC11 has an age-dependent, brain region specific function in regulating FEZ1, a gene associated with schizophrenia. This is important because it demonstrates that the regulatory role of HDAC11 in the hippocampus is developmentally regulated. Given previous associations between HDAC11, FEZ1 and schizophrenia, a goal of future research should be to examine the performance of homozygous *Hdac11* knockout mice in behavioural tests.

## Results

### HDAC11 Expression Increases with Neural Differentiation with Nuclear and Synaptic Localisation

**Figure 1:**
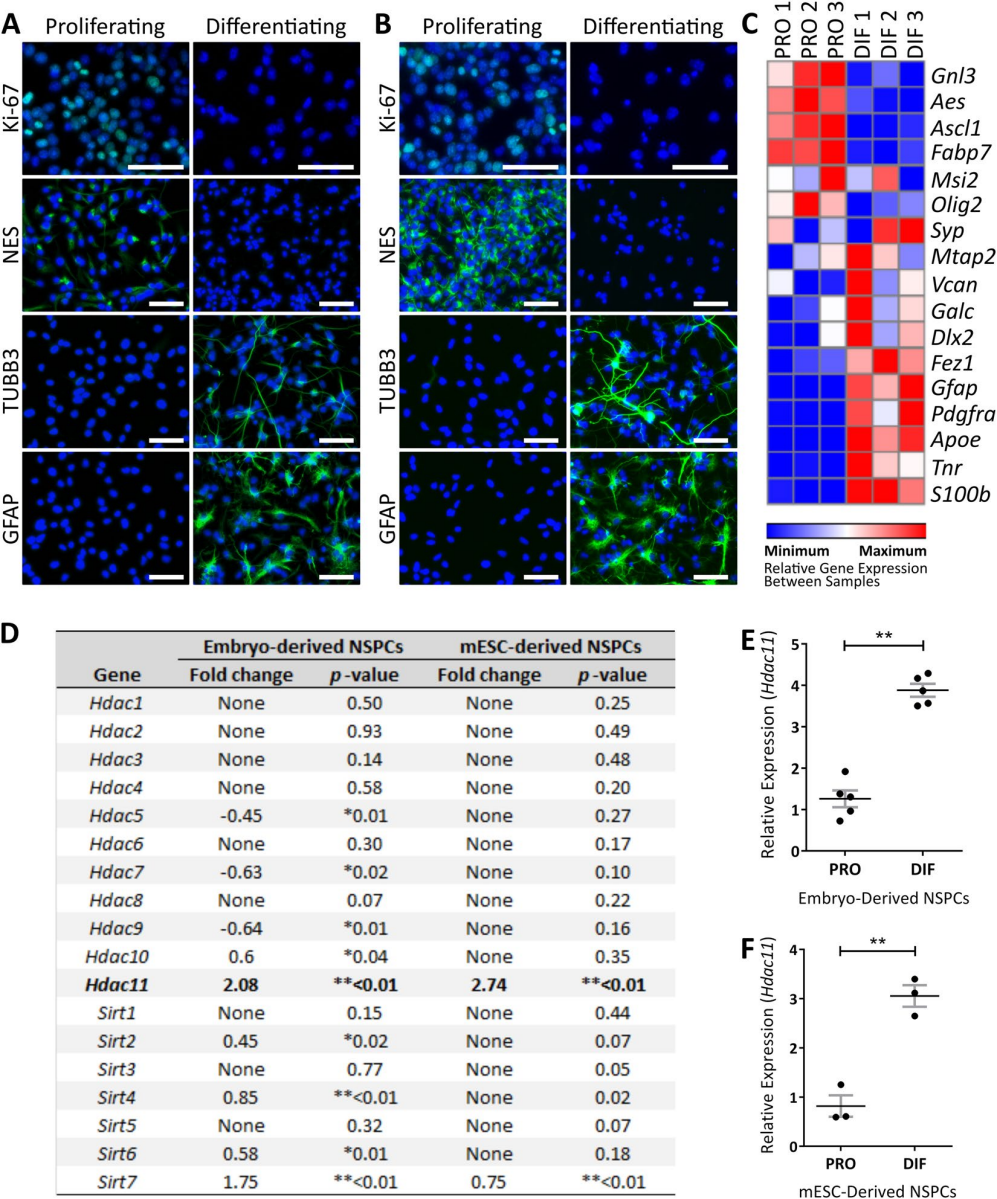
*Hdac11* displays the greatest and most consistent increase in expression as neural cells differentiate. Neural cells were derived from (**A**) the ganglionic eminence of E14.5 mouse embryos and (**B**) mESCs then examined for immunoreactivity of Ki-67, Nestin (NES), TUBB3, and GFAP antibodies following 3 days in either proliferation or differentiation inducing conditions. (**C**) Relative expression of genes associated with neural proliferation in mouse embryo-derived neural cells maintained in proliferative (PRO) conditions or following 3 days of differentiation (DIF) inducing conditions. The heat map displays relative expression values between samples (i.e. across rows). Gene expression was normalised to the mean of *Canx* and *Sdha*. The full names of the genes and assays used are listed in Supplementary Table 3. *Hdac11* expression increases in (**D**,**E**) E14.5 mouse embryo-derived (*N* = 3, dots = individual sample values, bars = mean ± *SE*, *p < 0.05 **p < 0.01, two-tailed paired Student’s *t*-test) and (**D**,**F**) mESC-derived neural stem/precursor cells (NSPCs) following 3 days of differentiation inducing conditions (*N* = 5, dots = individual sample values, bars = mean ± *SE*, *p < 0.05 **p < 0.01, one-tailed paired Student’s *t*-test). Gene expression was normalised to the geomean of *Ubc*, *Actb* and *Eif4a2*.

Neural cell lines were generated from the mouse ganglionic eminence (Fig. 1A) and mouse embryonic stem cells (mESCs) (Fig. 1B) to profile HDAC gene expression during differentiation. Following 3 days of differentiation inducing conditions there was a decrease in the expression of proliferation associated Ki-67 and neural stem/precursor cell associated nestin. In contrast, the expression of neuronal associated tubulin beta-3 chain (TUBB3) and astrocyte associated glial fibrillary acidic protein (GFAP) increased (Fig. 1A,B). This transition was further demonstrated by the changing profile of genes associated with neural proliferation and differentiation (Fig. 1C; Supplementary Fig. 1). Examination of *Hdac* gene expression levels between proliferating and differentiating neural cells revealed that *Hdac11* has the largest increase in expression compared to all the other *Hdacs* and *Sirtuins* (Fig. 1D; Supplementary Fig. 2). This is similarly observed in mouse embryo-derived and mESC-derived neural cells (Fig. 1D–F).

**Figure 2:**
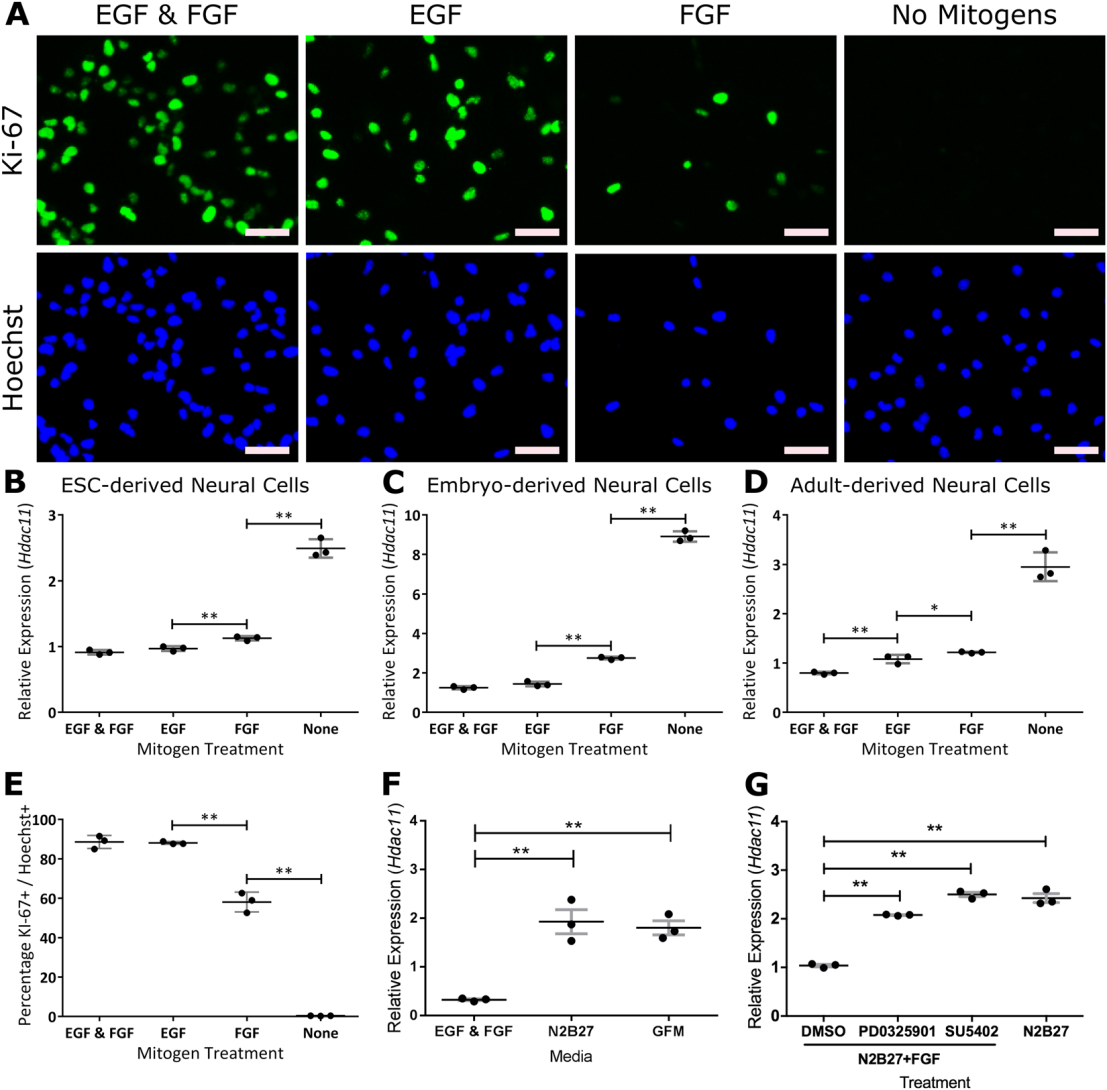
*Hdac11* expression responds to mitogen withdrawal from mESC-, embryonic mouse-, and adult mouse-derived neural cells. (**A**) Embryo-derived neural cells positive for proliferative marker Ki-62 following withdrawal of mitogens epidermal growth factor (EGF) and/or fibroblast growth factor (FGF) for 3 days. Expression of *Hdac11* in (**B**) embryo-derived neural cells, (**C**) mESC-derived neural cells and (**D**) adult mouse-derived neural cells increases following withdrawal of mitogens. (**E**) Percentage of proliferating cells in each condition as indicated by nuclear Ki-67 immunoreactivity. (**F**) *Hdac11* expression in media containing non-mitogenic growth factors BDNF, GDNF, NGF and NT-3 (collectively GFM “Growth factor media”). (**G**) Impact of small molecule inhibitors PD0325901 (1 µM) and SU5492 (10 µM) on *Hdac11* expression in the presence of FGF. *N* = 3, dots = individual sample values, bars = mean ± SE, **p < 0.01, *p < 0.05, one-tailed paired Student’s *t*-test. Gene expression was normalised to the mean of *Canx* and *Sdha*.

Increasing *Hdac11* expression was closely related to progressive removal of mitogens epidermal growth factor (EGF) and fibroblast growth factor (FGF). Removal of mitogens from the culturing media results in fewer proliferating cells (Fig. 2A,E). This mirrored the pattern of increasing *Hdac11* expression as the mitogens were removed from mESC-derived (Fig. 2B), embryonic mouse-derived (Fig. 2C) and adult mouse-derived (Fig. 2D) neural cells. When the growth factors that encourage proliferation (EGF & FGF) were replaced with growth factors that encourage differentiation (Brain-derived neurotrophic factor (BDNF), Glial-derived neurotrophic factor (GDNF), Nerve growth factor (NGF) & Neurotrophin-3 (NT-3)) there was an increase in *Hdac11* expression (Fig. 2F). Accordingly, small-molecule compounds that are reported to inhibit pathways associated with FGF signalling also increased the expression of *Hdac11* (Fig. 2G).

**Figure 3:**
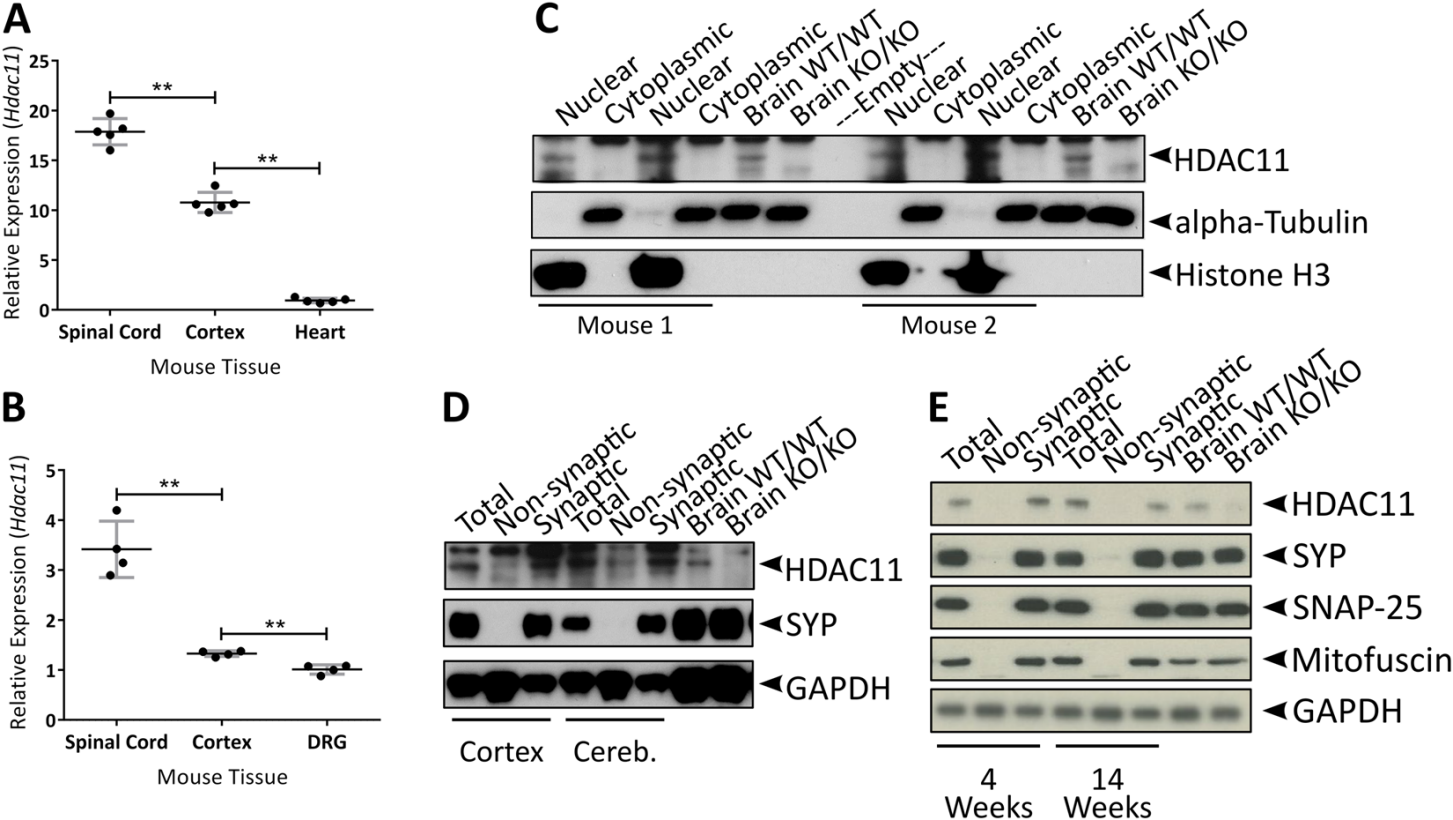
HDAC11 protein is enriched in the nucleus and at the synapse. (**A**) Expression of *Hdac11* in neural and non-neural tissue of 2-month-old mice. (**B**) Expression of *Hdac11* in tissue prepared from cortex, spinal cord and dorsal root ganglion tissue of P7 mice. (**C**) HDAC11 was enriched in the nuclear fraction but not in the cytoplasmic fraction of mouse brain lysate. (**D**) HDAC11 was enriched in synaptic fractions prepared from mouse cortex and cerebellum lysates. (**E**) HDAC11 was enriched in synaptic fractions prepared from the cortex of 4 week and 14 week-old mice (SYP = Synaptophysin; SNAP-25 = Synaptosomal-associated protein 25). *N* = 4–5, dots = individual sample values, bars = mean ± STD, **p < 0.01, two-tailed unpaired Student’s *t*-test.

*Hdac11* expression was comparatively high in the mouse brain relative to the other HDACs (Supplementary Table 6). Expression was highest in the spinal cord and all neural tissue displayed greater expression than the heart (Fig. 3A,B; Supplementary Fig. 3A). Examination of total brain lysates demonstrated that HDAC11 was enriched in the nuclear fractions (Fig. 3C; Supplementary Figs 3B, 4A,B). However, as there are reports of HDAC11 in the cytoplasm of neural cells^26,29^, we decided to look more closely at this compartment. Interestingly, HDAC11 was found to be enriched in synaptosomal preparations (Fig. 3D; Supplementary Figs 3C, 4C,D) at both 4 and 14 weeks of age (Fig. 3E; Supplementary Figs 3D, 5A–D). Unfortunately, the HDAC11 antibodies used in this study proved to have a lot of unspecific binding (Supplementary Figs 3 and 6). We assessed HDAC11 expression using western blotting with *Hdac11* ^KO/KO^ lysates as negative controls. However, we were unable to examine subcellular localisation with histological methods because *Hdac11* ^WT/WT^ and *Hdac11* ^KO/KO^ tissue displayed similar patterns of reactivity following the conditions used in this study (Supplementary Fig. 7).

### Characterisation of *Hdac11*^KO/KO^ Mice

**Figure 4:**
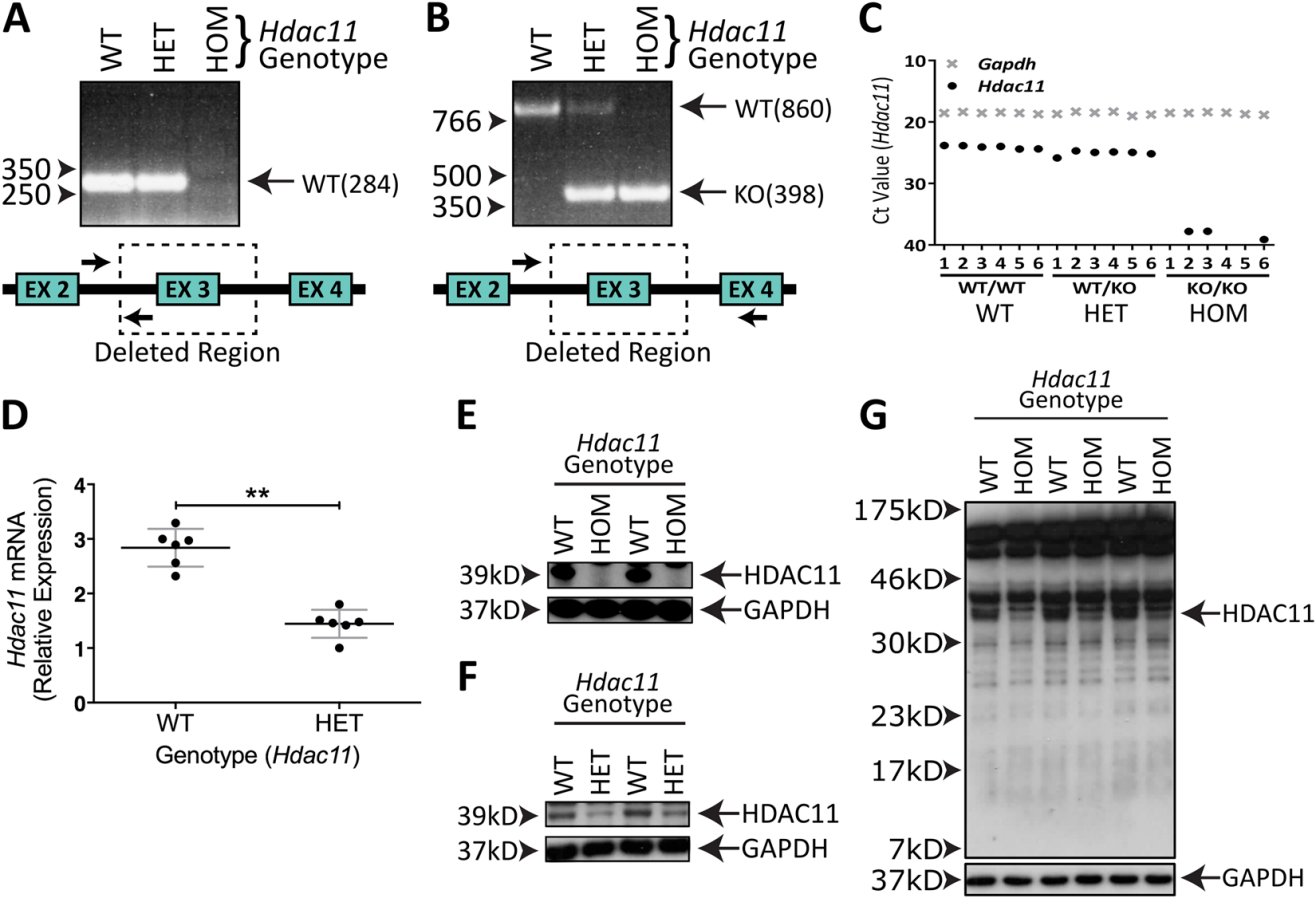
Mutation of *Hdac11* gene results in loss of HDAC11 expression. (**A**) A primer pair (arrows) requiring a sequence in the deleted region (dotted box) encompassing exon 3 of the Hdac11 gene fails to produce a 284 bp in *Hdac11* ^KO/KO^ (HOM) mice. (**B**) A primer pair that covers the deleted region yields a smaller product in mice heterozygous or homozygous for the mutant allele. (**C,D**) Lower levels of the *Hdac11* transcript and (**F) ** HDAC11 protein were detected in *Hdac11* ^WT/KO^ mice compared to *Hdac11* ^WT/WT^ mice. Gene expression was normalised to that of *Gapdh*. (**E**) No HDAC11 protein was detected in *Hdac11* ^KO/KO^ mice. (**G**) A truncated form of HDAC11 was not detected in *Hdac11* ^KO/KO^ mouse brain. Antibody used in panels 4E, 4F and 4G is anti-HDAC11 (Sigma, Catalogue #H4539). *N* = 6 (dots = individual sample values), bars = mean ± STD, **p < 0.01, one-tailed unpaired Student’s *t*-test.

**Figure 5:**
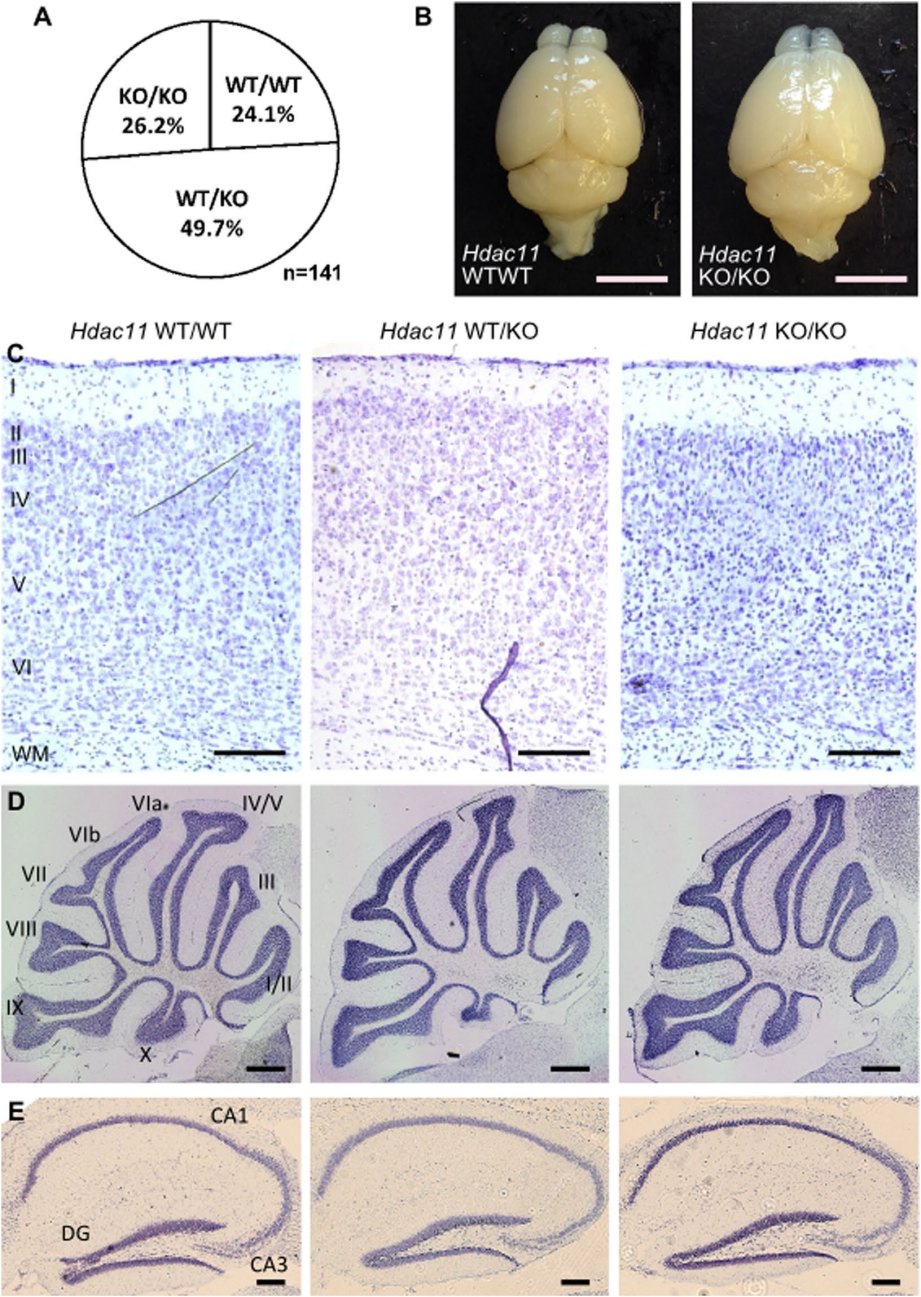
*Hdac11* ^KO/KO^ mice are born at Mendelian ratios with normal gross brain morphology. (**A**) Viable mice were born at approximately Mendelian ratios. (**B**) The gross brain morphology of two-month-old *Hdac11* ^KO/KO^ mice is similar to *Hdac11* ^WT/WT^ mice. Cresyl violet (Nissl) staining in (**C**) cortical (layers I-VI, WM = white matter) (**D**) cerebellar (lobules I-X) and (**E**) hippocampal regions (DG = Dentate Gyrus; CA = Cornu Ammonis). Scale bars = (**B**) 5 mm, (**C**) 200 µm (**D**) 500 µm and (**E**) 200 µm.

Mice with complete loss of exon 3 from the *Hdac11* gene (Fig. 4A,B; Supplementary Fig. 8A,B) were born at Mendelian ratios (Fig. 5A). Accordingly, no transcript was detected by primers complementary to the cDNA sequence within exon 3 (Fig. 4C). The *Hdac11* transcript was detected in brain lysates at reduced levels in *Hdac11* ^WT/KO^ mice compared to *Hdac11* ^WT/WT^ mice (Fig. 4D). There was no impact on the expression of the other HDAC transcripts (Supplementary Fig. 9). We examined HDAC11 protein using an antibody recognising the amino acids 2–16 at the N-terminus (Fig. 4E,F; Supplementary Figs 6A,E, 8C) and an antibody recognising a more C-terminal part of the protein at amino acid 269 (Supplementary Fig. 6B). *Hdac11* ^KO/KO^ mice displayed complete loss of the protein (Fig. 4E, Supplementary Figs 6A, 8C) whereas *Hdac11* ^WT/KO^ mice expressed HDAC11 at a level lower than wild type mice (Fig. 4F, Supplementary Figs 6F, 8D). The antibody targeting the N-terminus of HDAC11 did not detect a remaining protein fragment in the *Hdac11* ^KO/KO^ brain tissue (Fig. 4G; Supplementary Figs 6G, 8E).

Given reports that HDAC11 has roles in proliferation and our observations that HDAC11 was intimately linked with the proliferative status of neural cells we reasoned that loss of HDAC11 may impact development. However, the brains of *Hdac11* ^KO/KO^ mice appeared similar to those of *Hdac11* ^WT/WT^ mice at 2 months of age (Fig. 5B). The cortex and cerebellum were of a similar size and there was no obvious regional disruption (Fig. 5C,D). Similarly, there was no disruption to the appearance of the major hippocampal regions (Fig. 5E). For closer examination of neural proliferation and differentiation we derived neural cells from *Hdac11* ^KO/KO^ mice for *in vitro* investigations.

### Disrupted Fez1 Expression in the Hippocampus of Adult *Hdac11*^KO/KO^ Mice

**Figure 6:**
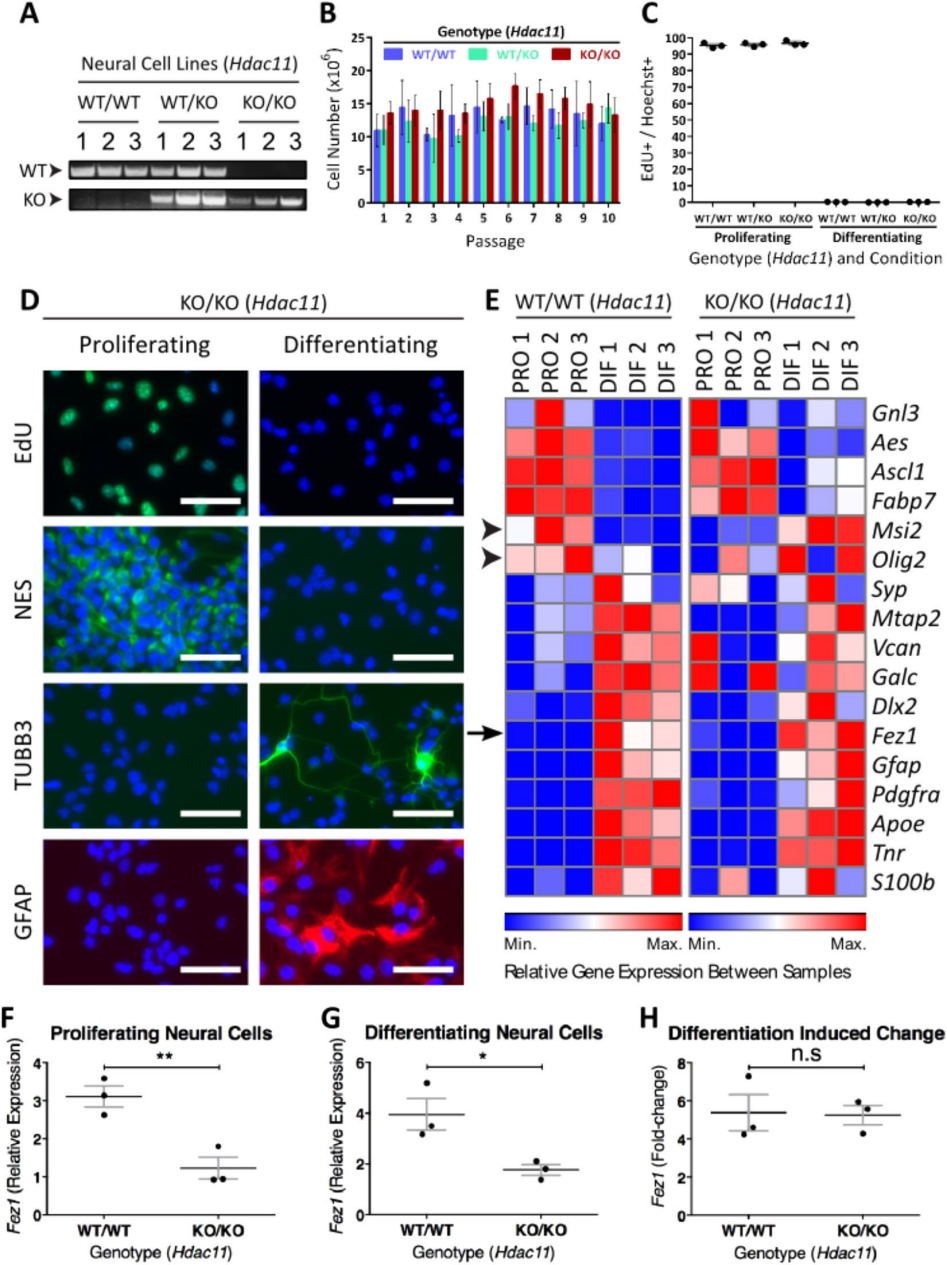
Reduced *Fez1* expression in neural cells derived from *Hdac11* ^KO/KO^ mice. (**A**) Three neural cell lines were generated from *Hdac11* ^WT/WT^, *Hdac11* ^WT/KO^ and *Hdac11* ^KO/KO^ mice. (**B**) Cells were counted following each passage every 4 days for 10 passages. (**C**) Cells derived from all mice exited the cell cycle following 3 days of differentiation inducing conditions. (**D**) Presence of EdU, Nestin (NES), TUBB3, and GFAP in cells derived from the ganglionic eminence of E14.5 *Hdac11* ^KO/KO^ mouse embryos following 3 days of proliferation or differentiation inducing conditions. (**E**) Relative expression of genes associated with neural proliferation in mouse embryo-derived neural cells given 3 days of either proliferation (PRO) or differentiation (DIF) associated conditions. *Msi2* and *Olig2* expression display opposing changes during the differentiation of *Hdac11* ^WT/WT^ and *Hdac11* ^KO/KO^ cells (arrow heads). The expression of *Fez1* (arrow) was decreased in neural cells derived from *Hdac11* ^KO/KO^ mice following 3 days of either (**F**) proliferating or (**G**) differentiating inducing conditions. (**H**) *Fez1* expression increased in neural cells derived from *Hdac11* ^WT/WT^ and *Hdac11* ^KO/KO^ mice at similar levels following 3 days differentiation. *N* = 3 (dots = individual sample values), bars = mean ± SE, **p < 0.01, *p < 0.05, two-tailed unpaired Student’s *t*-test. Gene expression was normalised to the mean of *Canx* and *Sdha*.

Neural cell lines generated from *Hdac11* ^*KO/KO*^ mice (Fig. 6A; Supplementary Fig. 8F) displayed similar proliferation rates to those generated from *Hdac11* ^WT/WT^ mice (Fig. 6B). 5-ethynyl-2′deoxyuridine (EdU) is a thymidine analogue which is incorporated into the DNA of dividing cells. All neural cells generated from *Hdac11* ^WT/WT^ and *Hdac11* ^*KO/KO*^ mice displayed no EdU incorporation over a 24 hour period following 2 days of induced differentiation (Fig. 6C). During this process the expression of neural stem/precursor cell and neural differentiation associated markers appeared largely normal (Fig. 6D,E; Supplementary Figs 10, 11, 12, 13). We observed similar levels of expression of all *Hdac* genes (except *Hdac11*) in *Hdac11* ^*KO/KO*^ *and Hdac11* ^*WT/WT*^ neural cell lines (Supplementary Fig. 9). Furthermore, the expression profile of neural associated genes was similar in neural cells generated from *Hdac11* ^*KO/KO*^ and *Hdac11* ^WT/WT^ mice (Fig. 6E). The exception being the expression of proliferation associated gene *Msi2* (Musashi 2) which displays a small decrease (−0.27-fold, p < 0.01) during the differentiation of *Hdac11* ^WT/WT^ cells but an increase (0.46-fold, p < 0.01) during the differentiation of cells derived from *Hdac11* ^*KO/KO*^ mice (Fig. 6E, arrow head). However, there was not a statistically significant difference in the level of *Msi2* expression when comparing *Hdac11* ^*KO/KO*^ and *Hdac11* ^WT/WT^ cells with each other in proliferating (p = 0.1) or differentiating (p = 0.24) conditions (Supplementary Table 7). *Olig2* displayed a similar pattern to *Msi2* (Fig. 6E, arrow head), and this too was not statistically different between *Hdac11* ^*KO/KO*^ and *Hdac11* ^WT/WT^ cells (Supplementary Table 7).

Comparison of gene expression profiles of proliferating neural cells derived from *Hdac11* ^*KO/KO*^ and *Hdac11* ^WT/WT^ revealed an impact on *Fez1* expression (Fig. 6F). *Fez1* expression increases following the differentiation of both *Hdac11* ^*KO/KO*^ and *Hdac11* ^WT/WT^ cells (Fig. 6E arrow, 6H). However, its expression remains lower in differentiating *Hdac11* ^KO/KO^ cells compared to *Hdac11* ^WT/WT^ cells (Fig. 6G). This was not due to a reduced increase of *Fez1* expression during the differentiation of *Hdac11* ^*KO/KO*^ neural cells (Fig. 6H). The impact on *Fez1* caught our attention because of Watanabe *et al*.’s reports of the regulatory activity of HDAC11 on FEZ1^26^. Additionally, it was the only gene on the gene expression panel to be affected in both proliferating and differentiating neural cells with statistical significance (Supplementary Table 7).

**Figure 7:**
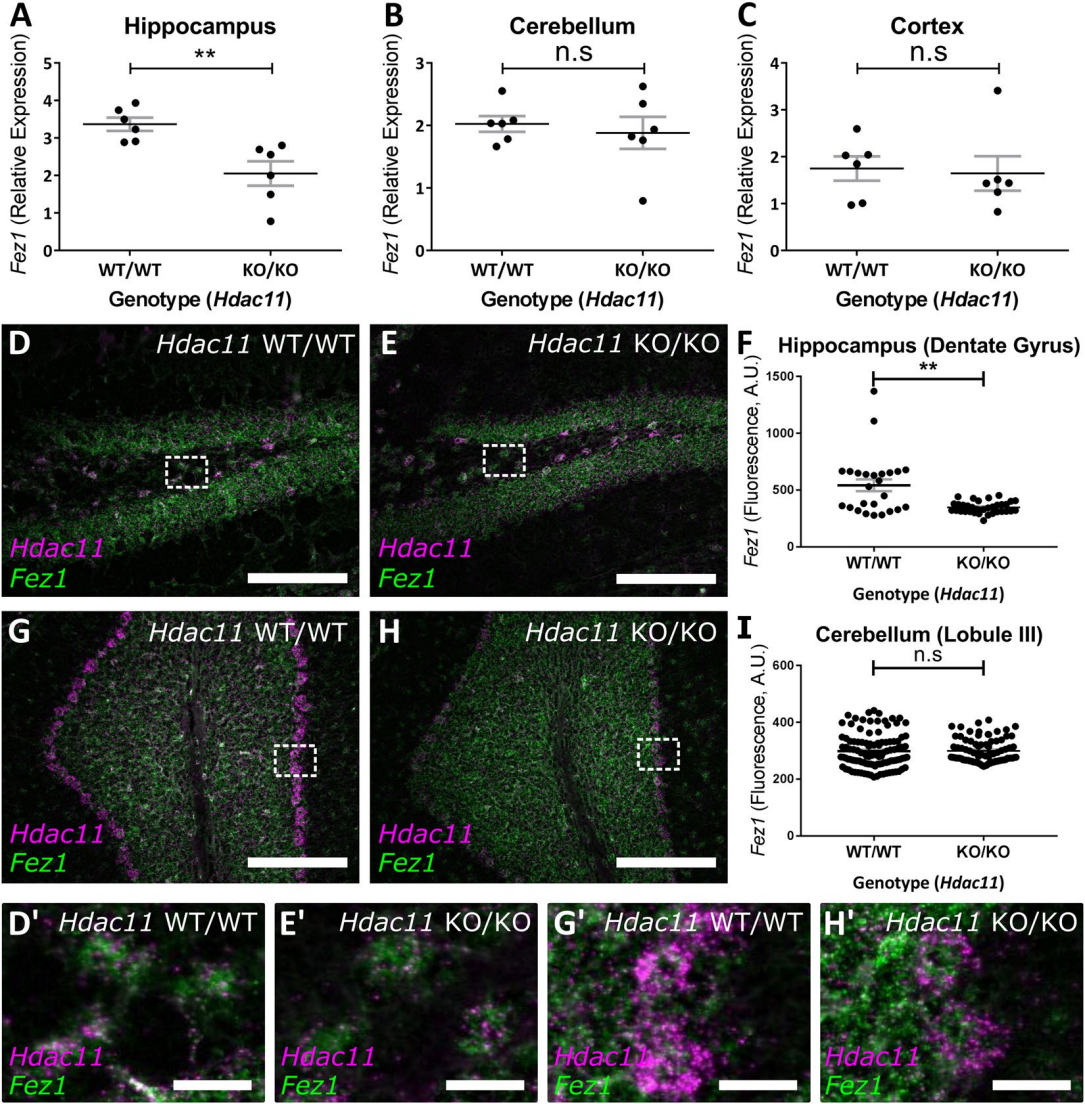
Reduced *Fez1* expression in the hippocampus of *Hdac11* ^KO/KO^ adult mice. The expression of *Fez1* was examined in the hippocampus, cortex, cerebellum and spinal cord of 2-month-old *Hdac11* ^KO/KO^ mice. (**A**) Only the hippocampus of *Hdac11* ^KO/KO^ mice had lower relative *Fez1* expression compared to *Hdac11* ^WT/WT^ mice. The expression of *Fez1* was unaffected in the (**B**) cerebellum or (**C**) cortex in *Hdac11* ^KO/KO^ mice. Gene expression was normalised to the mean of *Canx* and *Sdha* (**A**–**C**). (**D**–**F**) Decreased *Fez1* expression was observed in selected *Hdac11* positive cells within the dentate gyrus of 2-month-old mouse hippocampus. Regions indicated with a perforated box are displayed in panels D’ and E’. (**G**–**I**) There was no impact on *Fez1* expression in selected *Hdac11* positive cells within the lobule III of 2-month-old mouse cerebellum. Regions indicated with a perforated box are displayed in panels G’ and H’. *N* = 6 (**A**–**C**), *N* = 3(*n* ≥ 25) (**F**,**H**) (dots = individual sample values), bars = mean ± SE, **p < 0.01, two-tailed unpaired Student’s *t*-test. *Fez1* = Green, *Hdac11* = Magenta, scale bar = 200 µm (**D**,**E**,**G**,**H**) and 25 µm (**D’,E’,G’,H’**).

We sought to corroborate observations from our *in vitro* studies and therefore examined *Fez1* expression in brain lysates from *Hdac11* ^*KO/KO*^ mice. This did not reveal an impact of HDAC11 loss on *Fez1* expression (Supplementary Fig. 14). To explore the possibility that the loss of HDAC11 has a region specific impact on the expression *Fez1* we examined the cortex, hippocampus and cerebellum of *Hdac11* ^*KO/KO*^. These experiments were done on juvenile (P10/“10 day old”) and adult (P60/“2 month old”) mice. This revealed that *Fez1* expression was specifically downregulated in the hippocampus of adult *Hdac11* ^*KO/KO*^ mice (Fig. 7A). In contrast, expression of *Fez1* in the hippocampus was unaffected in juvenile *Hdac11* ^*KO/KO*^ mice (Supplementary Fig. 15A). This age-dependent effect on *Fez1* is region specific because it was expressed at similar levels in the cortex and cerebellum of *Hdac11* ^*KO/KO*^ juvenile (Supplementary Fig. 15B,C) and adult mice (Fig. 7B,C).

Using *in situ* hybridisation, we observed reduced *Fez1* expression in *Hdac11* positive cells within the hippocampus but not the cerebellum (Fig. 7D–I; Supplementary Fig. 15D,E). In contrast, *Hdac11* was reduced in both the hippocampus and the cerebellum (Supplementary Fig. 15F,G). Using qPCR, we confirmed the presence of an *Hdac11* transcript in *Hdac11* ^*KO/KO*^ tissue with an assay that targets the cDNA sequence which has not been deleted in the mutant. This assay detects RNA expression, albeit at lower levels in *Hdac11* ^*KO/KO*^ mice which is likely a result of nonsense mediated decay of the mutant transcript (Supplementary Table 8). Also for comparison, we examined oligodendrocyte associated genes *Mbp* (Myelin basic protein) and *Plp1* (Proteolipid protein 1) because they had previously been reported to be decreased following loss of *Hdac11* ^10^. However, we did not see a statistically significant impact on the expression of either of these genes (Supplementary Table 8).

## Discussion

Our observations are consistent with previous work demonstrating a particularly high abundance of HDAC11 in neural tissue^1,8,9^. Reports that its expression increases as neural cells differentiate were replicated using multiple *in vitro* approaches^10,11^. These observations may reflect emerging roles for HDAC11 as a regulator of the cell cycle and neural differentiation^10,12,22,23,26^. Previous studies had examined the consequence of partial loss or increase in HDAC11 expression^10,12,26^. However, the consequence of complete *Hdac11* knockout in a neural context had not yet been reported. The aim of this study was to examine the neural tissue of homozygous *Hdac11* knockout mice.

Fertile, homozygous *Hdac11* knockout mice were born at Mendelian ratios with no obvious phenotypes. This contrasts with the homozygous knockout mice of all class I HDACs (*Hdac1*, -*2*, -*3*, and -*8*) and certain class II HDACs (*Hdac4* and -*7*)^30,31^ which display either complete or partial embryonic/perinatal lethality^27,28^. *Hdac11* expression increases during the first 2 months of postnatal mouse brain development^9^. However, we found that the gross anatomy of the brain appeared normal in 2-month-old homozygous *Hdac11* knockout mice. Three of the previously generated *Hdac* knockout mice (*Hdac5*, -*6*, and -*9*) displayed only subcellular or age-dependent phenotypes^32–34^. Similarly, *Hdac11* knockout mice do not display an obvious phenotype but rather have an age dependent brain region specific decrease in *Fez1*. It is interesting to note that HDAC11 and HDAC6 appear to be recruited to the same protein complex^1,26^ and both are reported to regulate CDC20, a component of the pathway that controls FEZ1 ubiquitination^26,35^.

Partial loss of *Hdac11* expression has been reported to impact on the proliferation of fibroblasts *in vitro* ^12^. Together with reports of roles for HDAC11 in the cell cycle^22,23^ and its association with cancer and brain volume^13–20^ we predicted that there would be an impact on the proliferation rate of cells derived from the nervous system of homozygous *Hdac11* knockout mice. However, we did not find any effect from HDAC11 loss on neural proliferation and these cells also exited the cell cycle when directed. Accordingly, the gross brain morphology of homozygous *Hdac11* knockout mice appeared normal suggesting these processes had at least generally been regulated appropriately. One possibility is that HDAC11 has roles specific to cancer cells. Supporting this idea is a report that HDAC11 knockdown selectively impacts carcinogenic cell lines, leaving normal cell lines unharmed^20^.

Previous studies have reported that knockdown of *Hdac11* results in decreased expression of *IL-10* (Interleukin 10)^24^, *Mbp* (Myelin basic protein) and *Plp* (Proteolipid protein 1) genes^10^. Additionally, Deubzer *et al*. report unpublished results that HDAC11 represses the transcription of *Bmp4* (Bone morphogenetic protein 4) in a neuroblastoma cell line^20^. We found that the expression of oligodendrocyte associated genes *Mbp* and *Plp* were expressed at reduced levels in the adult *Hdac11* ^KO/KO^ hippocampus but this was not statistically significant. It is likely that loss of HDAC11 has cell type specific effects on gene expression which should be considered carefully when investigating its function. The previous studies on HDAC11 function had examined macrophages^24^, oligodendrocyte precursor cell lines^10^, fibroblast cells^12^ and a neuroblastoma cell line^20^. We propose that the *Hdac11* knockout mouse line can be used for investigation of tissues and cell types relevant to these experiments.

HDAC11 was initially reported to be localised to the nucleus^1^ where it is understood to regulate gene expression^10,24^. Indeed, the results of this study show that HDAC11 is enriched in nuclear fractions. It has been reported that HDAC11 becomes increasingly localised in cytoplasmic compartments during the differentiation of neural progenitors in the hippocampus^26^. Despite limitations preventing us using HDAC11 antibodies to examine HDAC11 localisation using immunocytochemistry, we did observe HDAC11 expression in cytoplasmic fractions and more specifically in synaptosomes. This provides additional insight into the subcellular localisation of HDAC11 and offers the possibility that it has a regulatory role in the synaptic compartment. Like class IIa HDACs, HDAC11 may shuttle between cellular compartments in response to stimuli. HDAC11 has previously been reported to regulate dendrite development^26^ and enrichment of HDAC11 at the synapse supports the enticing possibility that regulating its activity will impact synaptic plasticity and memory.

We observed decreased *Fez1* expression in both proliferating and differentiating cells derived from homozygous *Hdac11* knockout mice so we investigated this in more detail. Examination of different brain regions revealed that this effect was specific to the hippocampus of adult mice which was surprising given that we also observed decreased *Fez1* expression in proliferating neural cells *in vitro*. This may reflect an acquired identity of these *in vitro* lines and reinforces the importance of follow up studies on appropriate tissue.

A previous study showed that FEZ1 ubiquitination is downstream of a pathway activated by HDAC11 activity^26^. Given their results, it would be predicted that loss of HDAC11 activity would increase FEZ1 protein expression. Regrettably, we do not report FEZ1 protein levels in response to loss of HDAC11 as we were unable to detect the expression of FEZ1 using commercially available antibodies. The decreased level of *Fez1* transcript in response to loss of HDAC11 may be due to a self-regulatory function of FEZ1. This self-regulatory function may involve interaction with DISC1 (Disrupted in schizophrenia 1)^36^. Patients with schizophrenia carrying a high risk allele for the disease in the *DISC1* gene show reduced *FEZ1* expression in the hippocampus^37^.

The report of reduced *FEZ1* gene expression in the hippocampus of patients with schizophrenia is particularly relevant as this was seen in the homozygous *Hdac11* knockout mice^37^. The relationship between HDAC11 activity and schizophrenia-associated phenotypes is strengthened by Watanabe *et al*.’s evidence for a signalling cascade implicating HDAC11, FEZ1 and DISC1^26^. Sheng *et al*. examined the CA1 and CA2/3 regions of the hippocampus from post-mortem schizophrenia patient tissue^38^. They identified copy number increases in the *HDAC11* gene which resulted in increased *HDAC11* expression specifically in the CA2/3 region. In contrast, post-mortem tissue from patients with a different psychiatric disease (bipolar disorder) displayed no difference in the copy number or expression of *HDAC11* compared to healthy controls. A more recent study reports an association between schizophrenia and single nucleotide polymorphisms (SNPs) in the human *HDAC11* gene^39^.

The emerging association between HDAC11 and schizophrenia is interesting as it should be considered during the development of therapeutic drugs. The promise of treating schizophrenia by selective inhibition of certain HDACs has previously been discussed in a review by Weïwer *et al*.^40^. In their review they note that *Hdac11* is expressed in the hippocampus and such patterns provide a spatial association with schizophrenia but emphasise that functional inferences cannot be drawn from this. The results of this study demonstrate that *Hdac11* knockout mice provide a means to examine the functional association between *HDAC11* and schizophrenia-associated *FEZ1*.

Our findings are specific to the adult-mouse hippocampus, suggesting that this area will be particularly important to following investigations, particularly for investigating the molecular pathways regulated by HDAC11. Disrupted performance on hippocampal dependent tasks such as the Morris water maze, T-maze and radial arm maze are reported in mouse models of schizophrenia^41^. Furthermore, it would be interesting to investigate the response of *Hdac11* knockout mice to phencyclidine, which is reported to result in a variety of phenotypes associated with schizophrenia including hyperlocomotion^41^. *Hdac11* knockout mice may share similar characterisics to *Fez1* knockout mice which do not display any anatomical abnormalities but do display a hyperlocomotion phenotype in a variety of tasks^42^.

Genetics is important in the etiology of schizophrenia but it remains difficult to determine the precise causative genetic factors in this complex polygenic disorder^43^. HDAC11 may have a more general association with psychiatric disease. Studies have found links between HDAC11, the administration and reinforcement of drugs. An impact on *Hdac11* expression is reported in the brain of rodents administered with cocaine^44^, alcohol^45^ and methamphetamine^46,47^. Incidentally *Fez1* ^KO/KO^ mice have an enhanced response to methamphetamine treatment, at least in part due to increased mesolimbic dopaminergic transmission^42^. Understanding how these observations converge on HDAC11 may provide an important insight into the neural mechanisms disrupted in a variety of disorders. Furthermore, it is important that we understand the consequences of losing HDAC11 activity as this will be valuable for guiding the development of isoform specific HDAC inhibitors as therapeutics for disease.

## Methods

### Cell culture media

Mouse Embryonic Stem Cell (mESC) proliferation media was formulated in a basal media of Dulbecco’s modified Eagle medium (DMEM), High Glucose, GlutaMAX™ (Life Technologies, Catalog #61965-026) with 20% embryonic stem cell grade FBS (Source BioScience LifeSciences, Catalog #S0451). The media contained 1× Non-Essential Amino Acids (Life Technologies, Catalog #11140-050), 50 μM beta-mercaptoethanol (Life Technologies, Catalog #31350-010) and 10 μg/mL (Leukemia inhibitory factor) LIF (Millipore, Catalog #ESG1106).

Neural proliferation media was formulated in a NeuroCult™ NSC Basal Medium (Mouse) (STEMCELL Technologies, Catalog #05700) with NeuroCult™ NSC Proliferation Supplement (Mouse) (STEMCELL Technologies, Catalog #05701). The medium contained 20 ng/mL recombinant mouse epidermal growth factor (rmEGF) (Peprotech Inc, Catalog #315-09) and 10 ng/mL recombinant mouse fibroblast growth factor (rmFGF) (Peprotech Inc, Catalog #450-33).

Neural differentiation media (N2B27) is a formulation of 50% DMEM/F-12, GlutaMAX™ (Life Technologies, Catalog #10565-018) and 50% Neurobasal® Medium (Life Technologies, Catalog #21103-049). This contained 0.5x N-2 Supplement (Life Technologies, Catalog #17502-048), 0.5x B-27® Serum-free Supplement (Life Technologies, Catalog #17504-044), 0.5x GlutaMAX™ Supplement (Life Technologies, Catalog #35050-087), 1x Non-Essential Amino Acids (Life Technologies, Catalog #11140-050) and 50 μM beta-mercaptoethanol (Life Technologies, Catalog #31350-010).

### Generation and expansion of mESC-derived neural cells

Generation of mESC-derived neural cells was carried out according to Conti *et al*.^48^ before they were maintained in the neural proliferation media described above.

### Isolation and expansion of mouse embryo-derived neural cells

The mouse embryonic ganglionic eminence was dissected and cultured according to Chojnacki and Weiss’s protocol^49^. The cells were cultured in suspension on untreated plastic in neural proliferation media (see cell culture media). They were passaged every 4 days by dissociation with Accutase® (Sigma, Catalog #A6964) and reseeded at a density of 10,000 cells/cm2.

### Experimental proliferation and differentiation of neural cell cultures

For consistency between approaches, all proliferating neural cell lines were seeded as an adhered monolayer on 0.1 mg/mL poly-DL-ornithine (Sigma, Catalog #P8638) followed by 5 μg/mL laminin (Life Technologies, Catalog #23017-015) coated plastic before experimental procedures began. Following a typical passage, proliferating neural cells were dissociated into a single cell suspension ensured by passing the suspension through a sterile 40 μm cell strainer (SLS, Catalog #352340) into a 50 mL tube. Cells destined for proliferative conditions were seeded at 20,000/cm^2^, whereas those for differentiation inducing conditions were seeded at 100,000/cm^2^. After 24 hours, the cells had adhered to the surface and the media was replaced with the experimental media. For proliferative conditions, the media was replaced with neural proliferation media (see cell culture media). For differentiation inducing conditions, the media was replaced with neural differentiation media (see cell culture media). The media was replaced with fresh media every day until the cells were harvested.

### 5-ethynyl-2’-deoxyuridine (EdU) labelling

Incorporation of the thymidine analogue EdU into the DNA of proliferating cells over a 24 hour period was detected using the Click-IT® EdU Alexa Fluor® 488 Imaging kit (Life Technologies, Catalog #C10337) according to the manufacturer’s instructions.

### Immunocytochemistry

Cells for immunocytochemistry assays were seeded into black walled and optical bottomed 96 well plates (Fisher Scientific, #10281092). They were fixed for 20 minutes in a 4% formaldehyde solution (Sigma, Catalog #F1635). Primary and secondary antibodies were prepared in Phosphate buffered solution (PBS) (Life Technologies, Catalog #14190-169) made with 10% donkey serum (Sigma, Catalog #D9663), 0.1% Triton-X 100 (Sigma, Catalog #T8717) as described in supplementary Table 1. After each step the samples were washed three times with PBS. All assays were counterstained for 10 minutes with Hoechst 33342 (Life Technologies, Catalog #H3570) prepared in PBS to a concentration 1 μg/mL.

### Microscopy and Image Analysis

Fluorescent images were acquired using an inverted microscope (Axio Observer, ZEISS). Analyses of Ki-67 or EdU positive cells were quantified using Fiji/ImageJ (http://imagej.nih.gov/ij/). Cell nuclei were set as regions of interest (ROI) as defined by the nuclear counter stain (i.e. Hoechst 33342). Then the average signal intensity (i.e. Ki-67 and EdU) was measured for each ROI using the Analyze Particles command. A cell was considered positive if the average signal intensity for its ROI was greater than the ROI with the maximum value from staining with the secondary antibody alone (Ki-67 experiment) or differentiation inducing (i.e. proliferation preventing) condition (EdU experiment).

### RNA Extraction and Real Time PCR

Real time PCR was carried out using the 2^−ΔΔCt^ method as described previously^50^. The reverse transcription was carried out using the High-capacity RNA-to-cDNA^TM^ Kit (Life Technologies, Catalog #4387406) according to the manufacturer’s instructions. TaqMan® assays (Supplementary Tables 2, 3, 4) and TaqMan® Gene Expression Master Mix (Life Technologies, Catalog #4369016) were used according to the manufacturer’s instructions. Two Hdac11 TaqMan® assays were used in this study as one covered the exon deleted in the Hdac11 mice *Hdac11* ^KO/KO^ (Exons 2–3; Mm01183513_m1) and the other does not (Exons 5–6; Mm00523422). Data were collected on the C100TM Thermal Cycler and CFX96TM Real-Time System (BIO-RAD). Data collection from TaqMan® Low Density Arrays (TLDA) was carried out on the ABI PRISM 7900HT Sequence Detection System (Applied Biosystems).

### Preparation of nuclear, cytoplasmic and synaptosome fractions

Nuclear and cytoplasmic fractionation was carried out as described in Davies *et al*.^51^. Synaptosome fractions were prepared as described in Valencia *et al*.^52^.

### Western Blotting

Western blotting was carried out as described by Landles *et al*.^53^. The antibody incubation details are described in Supplementary Table 5. Following washes with PBS/0.1% Tween® 20 to remove residual antibody, the membranes were developed using an Amersham ECLTM Western Blotting Kit (GE Lifesciences, Catalog #RPN2109) and Amersham Hyperfilm ECLTM (GE Lifesciences, Catalog #28906837).

### *Hdac11* knockout mice

Details of the *Hdac11* knockout mice can be found on the Taconic website (http://www.taconic.com/mouse-model/hdac11-ko-6978 ). The gene was targeted in C57BL/6 ESCs by deletion of exon 3 leading to a premature stop codon. To identify the genotype, a PCR reaction with the forward primer (TGCTGCCTGTGAGCCACTGC) and reverse primer (AGAATGGCTGTCTCCCTAGG to detect the wild type allele; or CCTTGGAATAGCATCTCAGG to detect the knockout allele) were used. The reaction was run on a program of 4 minutes at 95 °C, before 36 cycles of 25 seconds at 95 °C, 20 seconds at 60 °C and 45 seconds at 72 °C. The program finished with a single final step for 6 minutes at 72 °C.

### Nissl staining

Mice were anesthetised with an injection of 100 μL sodium pentobarbital (J.M.L, Catalog #103-9130). The mice were transcardially perfused with 20 mL of PBS followed by perfusion with 20 mL of 10% formalin (i.e. 4% PFA) (PRC, Catalog #PRC/R/38). After the perfusion was complete, the brain was stored in 10% formalin at 4 °C overnight before equilibration in 30% sucrose (Fisher Scientific, Catalog #10744771) made in PBS at 4 °C. The brain was then snap frozen in 2-methylbutane (Sigma, Catalog #277258) that had been cooled on dry ice. The brains were sectioned every 30 μm on a cryostat, cooled to −17 °C. The sections were attached to glass adhesion SuperFrost® plus slides (VWR, Catalog #631-0446). They were stained in a 1% cresyl violet (Acros Organics, Catalog #405760025), 1% Glacial acetic acid solution for 10 minutes at room temperature (cerebellum and hippocampus images) or 60 minutes at 37 °C (cortex images). The slides were then dehydrated in increasing amounts of ethanol with only a few seconds incubation in the final 100% ethanol step to avoid removing too much stain. They were then incubated in Xylene (Sigma, Catalog #33817) for 15 minutes before mounting with DPX mounting medium (Fisher, Catalog #D/5319/05).

### *In situ* hybridisation

*In situ* hybridisation was carried out using RNAscope® Technology (Advanced Cell Diagnostics) according to the manufacturer’s instructions. The *Hdac11* probe targeted the region 35-981 of NM-1449.9.2. The *Fez1* probe targeted the region 2-1010 of NM-183171.4. To ensure that comparative numbers of cells were included in the quantification, single, individual cells expressing *Hdac11* were selected within the region of interest (dentate gyrus of the hippocampus) (see Supplementary Fig. 15D,E).

## Electronic supplementary material

Supplementary Information
