# Supplementary material for: Disruption to schizophrenia-associated gene Fez1 in the hippocampus of HDAC11 knockout mice
Source: Sci Rep. 2017 Sep 19;7:11900. doi: 10.1038/s41598-017-11630-1 (PMC5605701; doi:10.1038/s41598-017-11630-1)
Supplement: Supplementary file 1 — Supplementary Information [file 41598_2017_11630_MOESM1_ESM.pdf]

Supplementary Information

Disruption to schizophrenia-associated gene *Fez1* in the hippocampus of HDAC11 knockout mice

Dale T. Bryant[1,2], Christian Landles[1], Aikaterini S. Papadopoulou[1], Agnesska C. Benjamin[1], Joshua K. Duckworth[2], Thomas Rosahl[3], Caroline L. Benn[2]* and Gillian P. Bates[1]*

[1]UCL Huntington's Disease Centre, Sobell Department of Motor Neuroscience, UCL Institute of Neurology, University College London, London, United Kingdom

[2]Neusentis, Pfizer Ltd, The Portway, Granta Park, Abington, Cambridge, United Kingdom

[3] Merck & Co., Inc., Kenilworth, NJ, USA

Supplementary Figure 1

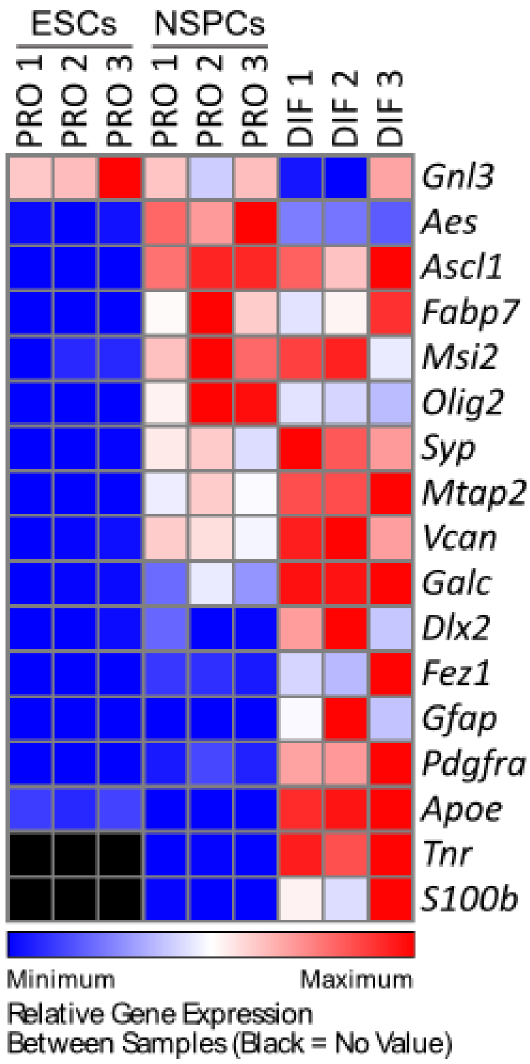

Supplementary Figure 1: Expression profile of neural associated genes as mESCs were induced to acquire a maturing neural phenotype. Relative expression of genes associated with neural proliferation in mESC-derived neural cells between proliferating mESCs (ESC, PRO), subsequently derived proliferating neural cells (NSPC, PRO) and finally following 3 days of differentiation (DIF) inducing conditions. The heat map displays relative expression values between samples (i.e. across rows). Gene expression was normalised to the mean of *Canx* and *Sdha*.

Supplementary Figure 2

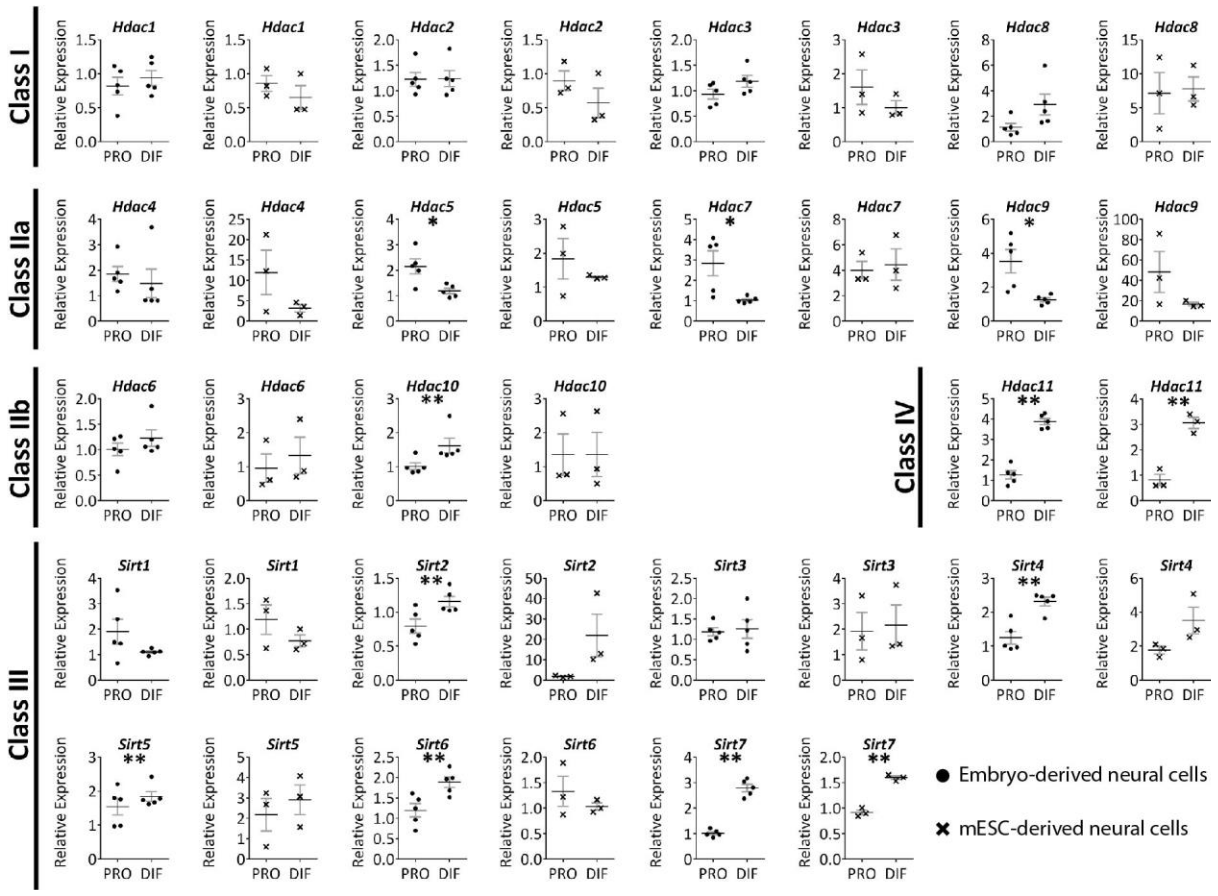

Supplementary Figure 2: Expression pattern of all *Hdacs* during *in vitro* neural differentiation. The expression of all *Hdac* genes were examined during the transition from proliferating neural cells to differentiating neural cells. This expression pattern is displayed for both E14 mouse embryo-derived neural cells (circles) and mESC derived neural cells (crosses). Neural cells had either been maintained in proliferation inducing NSC media (PRO) or differentiation inducing N2B27 media (DIF) for 3 days. For mESC-derived neural cells: $N = 3$; for E14.5 mouse-derived neural cells: $N = 5$, circles/crosses = individual sample values, bars = mean ± SE, *$p<0.05$, **$p<0.01$, two-tailed paired Student's *t*-test (embryo-derived neural cells), one-tailed paired Student's *t*-test (mESC-derived neural cells). Gene expression was normalised to the geomean of *Ubc*, *Actb* and *Eif4a2*.

Supplementary Figure 3

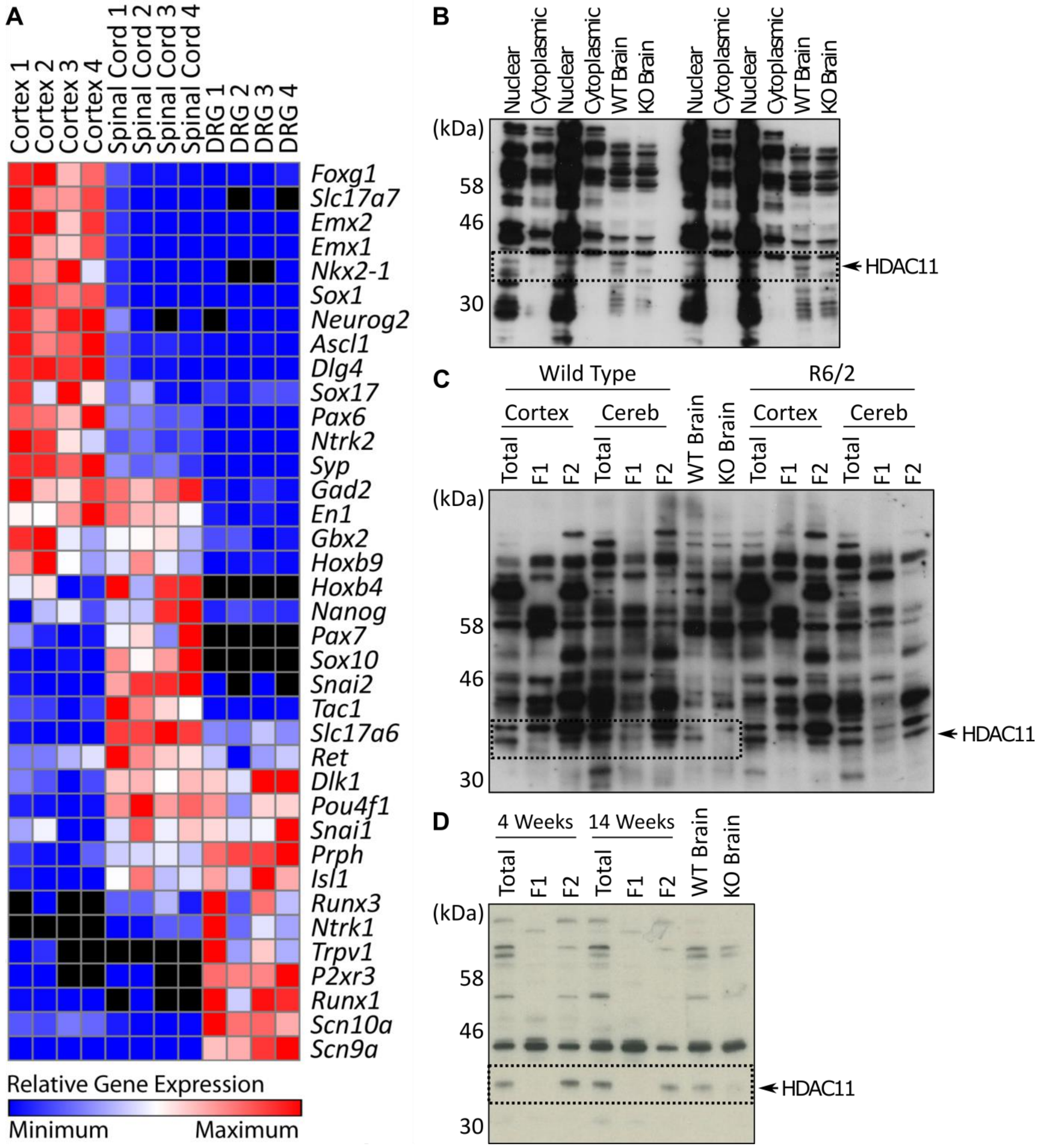

Supplementary Figure 3: Gene expression profile in dissected tissue and full display of HDAC11 western blots loaded with nuclear, cytoplasmic and synaptic fractions. A) Validation of mouse dorsal root ganglion (DRG) dissections by gene expression profile. The full names of the genes and assays used are listed in Supplementary Table 4. Gene expression was normalised to the mean of *Canx* and *Sdha*. **B)** Immunoreactivity of HDAC11 antibody (Sigma, Catalog #H4539) on nuclear and cytoplasmic fractions from mouse brain (Corresponds to Fig. 3C). **C)** Immunoreactivity of HDAC11 antibody (Sigma, Catalog #H4539) on fractions isolated during the enrichment of synaptic proteins from mouse cortex and cerebellum (Corresponds to Fig. 3D). **D)** Immunoreactivity of HDAC11 antibody (Abcam, Catalog #Ab18973) on fractions isolated during the enrichment of synaptic proteins from mouse cortex at 4 and 14 weeks of age (Corresponds to Fig. 3E). Dotted boxes indicate location of cropped images displayed in Fig. 3.

Supplementary Figure 4

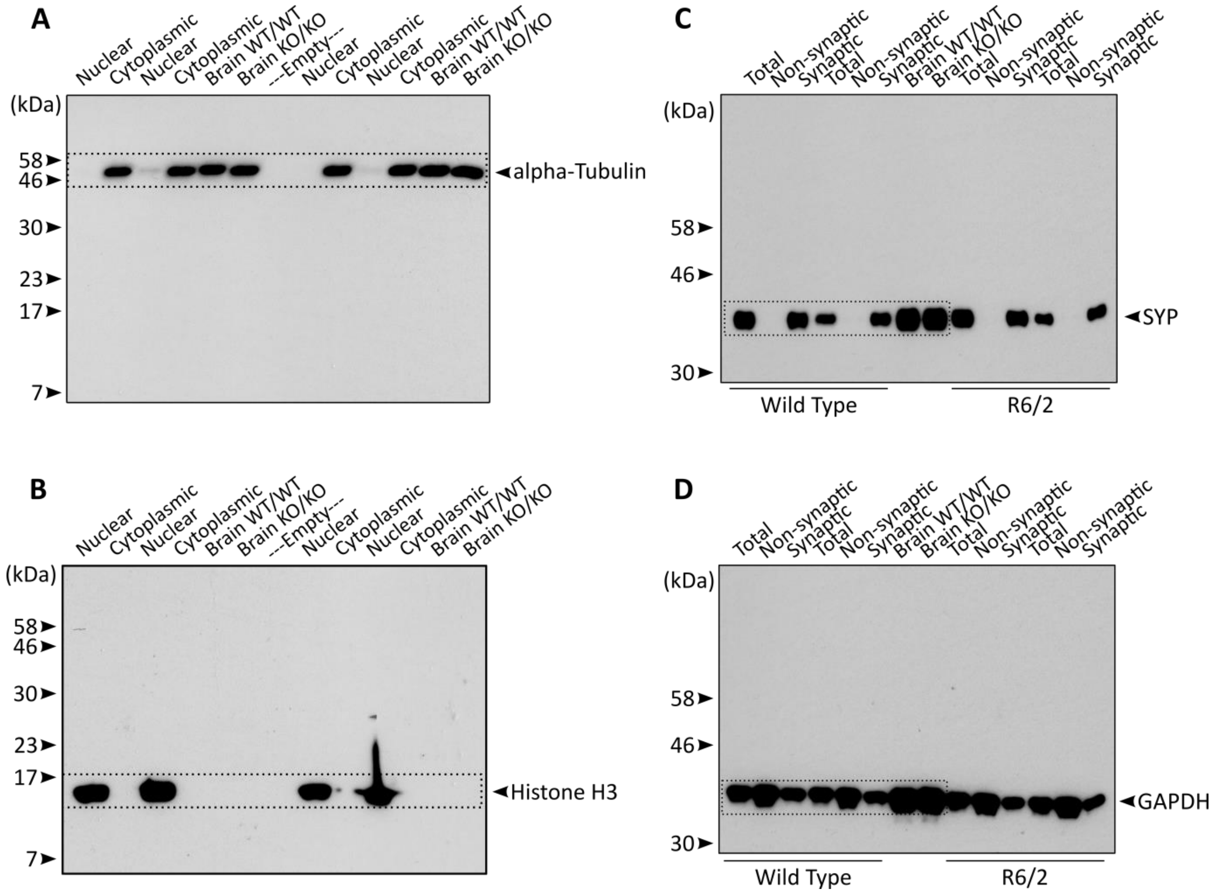

Supplementary Figure 4: Whole western blots for reference proteins displayed in Fig. 3C and Fig 3D. Perforated boxes indicate location of cropped images.

Supplementary Figure 5

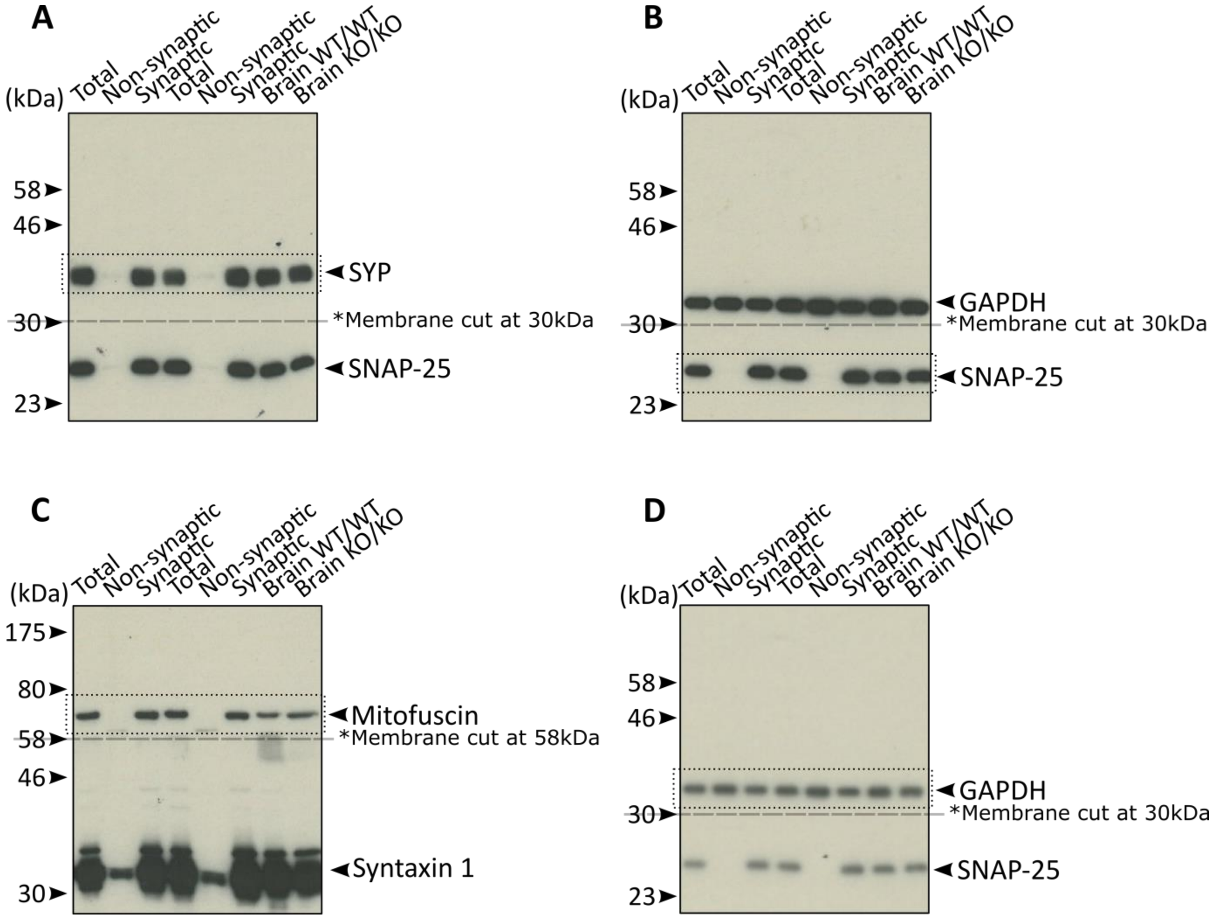

Supplementary Figure 5: Whole western blots for reference proteins displayed in Fig. 3E. Perforated boxes indicate location of cropped images. *Membranes were cut as indicated and probed with the annotated antibodies.

Supplementary Figure 6

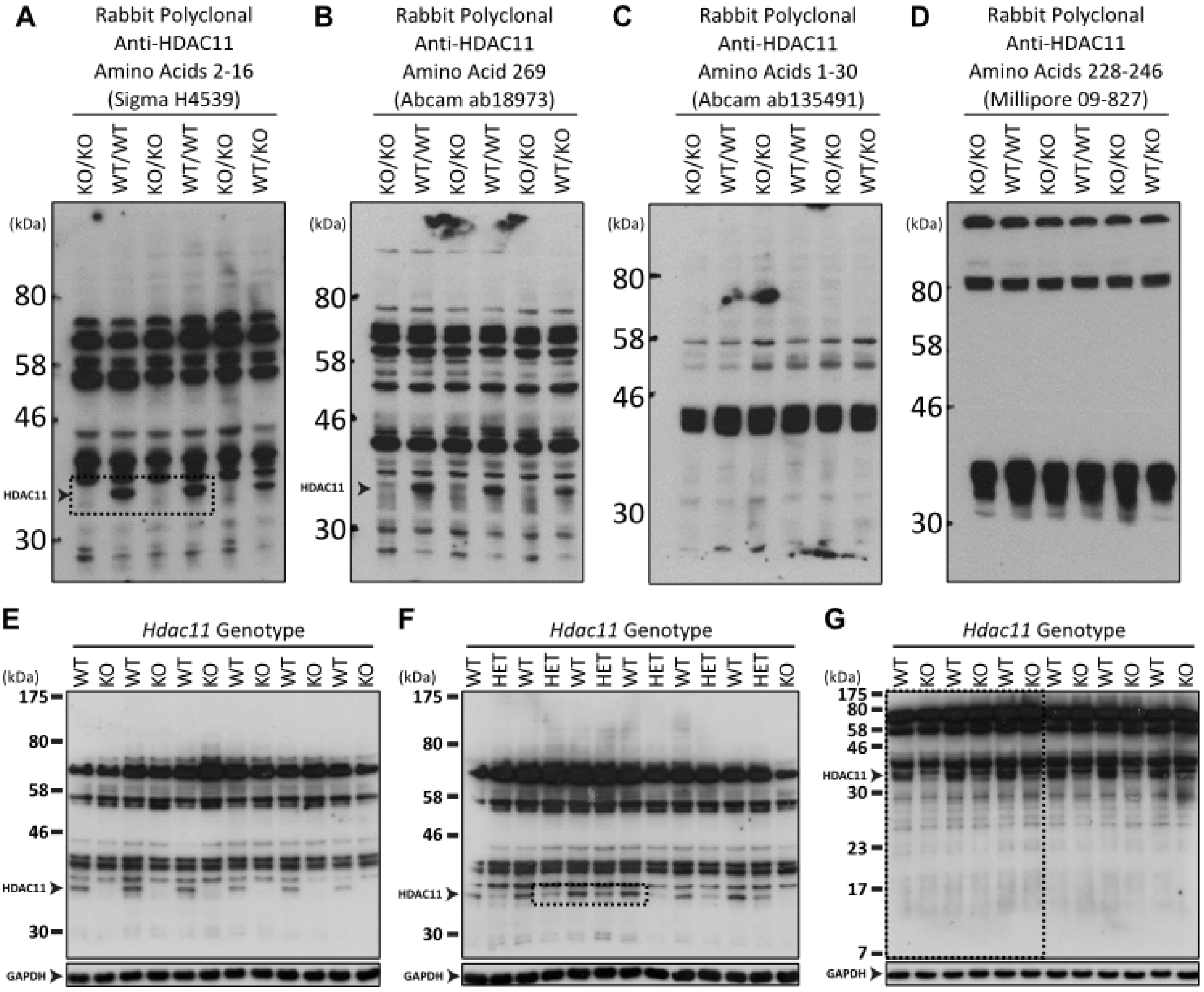

Supplementary Figure 6: Immunoreactivity of HDAC11 antibodies on *Hdac11*$^{KO/KO}$ brain tissue. Immunoreactivity of HDAC11 antibodies **A)** Sigma, Catalog #H4539 (Corresponds to Figure 4E), **B)** Abcam, Catalog #Ab18973, **C)** Abcam, Catalog #Ab135491, and **D)** Millipore, Catalog #09-827 on *Hdac11*$^{WT/WT}$, *Hdac11*$^{KO/KO}$ and *Hdac11*$^{WT/KO}$ mouse brain lysates. Full display of western blot loaded with **E)** *Hdac11*$^{WT/WT}$ and *Hdac11*$^{KO/KO}$ mouse brain lysates; **F)** *Hdac11*$^{WT/WT}$ and *Hdac11*$^{WT/KO}$ mouse brain lysates (Corresponds to Fig. 4F); and **G)** *Hdac11*$^{WT/WT}$ and *Hdac11*$^{KO/KO}$ mouse brain lysates blotted with retained low molecular weight protein on the gel (Corresponds to Fig. 4G). Antibody used in panels 4E and 4F is anti-HDAC11 (Sigma, Catalogue #H4539). Dotted boxes indicate location of cropped images displayed in Fig. 4.

Supplementary Figure 7

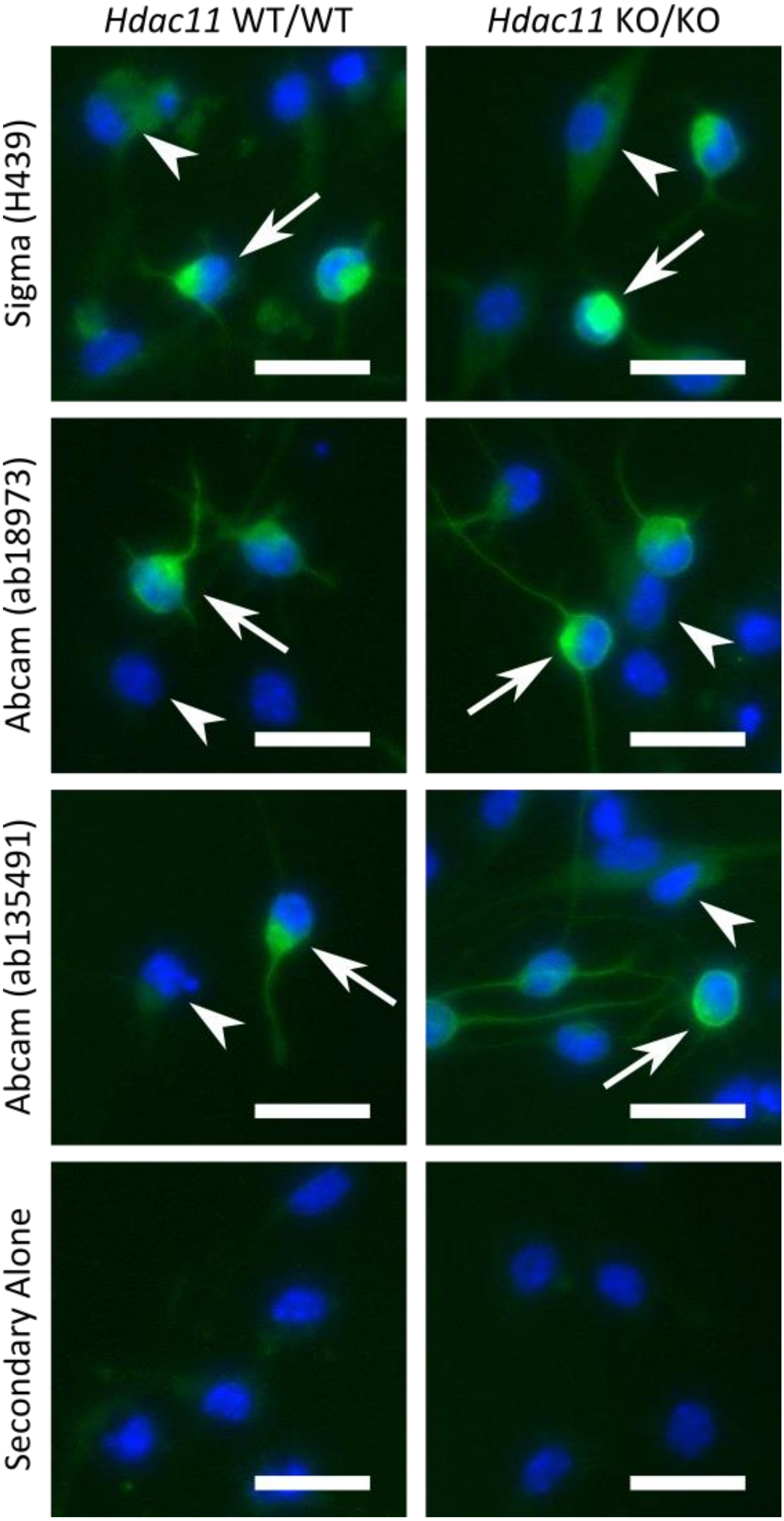

Supplementary Figure 7: Immunoreactivity of HDAC11 antibodies examined by fluorescent microcopy. Immunoreactivity of HDAC11 antibodies with mouse-derived neural cells following 4 days of differentiation as assessed by fluorescent microscopy. The pattern of immunoreactivity with each antibody was similar in *Hdac11*$^{WT/WT}$ and *Hdac11*$^{KO/KO}$ neural cell populations. A subpopulation of cells with neuronal morphology were positive for immunoreactivity with all HDAC11 antibodies in both *Hdac11*$^{WT/WT}$ and *Hdac11*$^{KO/KO}$ neural cells (arrows). Many cells that typically had fibrous morphology displayed little to no immunoreactivity with the HDAC11 antibody in both *Hdac11*$^{WT/WT}$ and *Hdac11*$^{KO/KO}$ neural cells (arrow heads). Green Signal = Immunoreactivity of HDAC11 antibodies, Blue signal = Hoechst/Nuclei, Scale bar = 20 µm.

Supplementary Figure 8

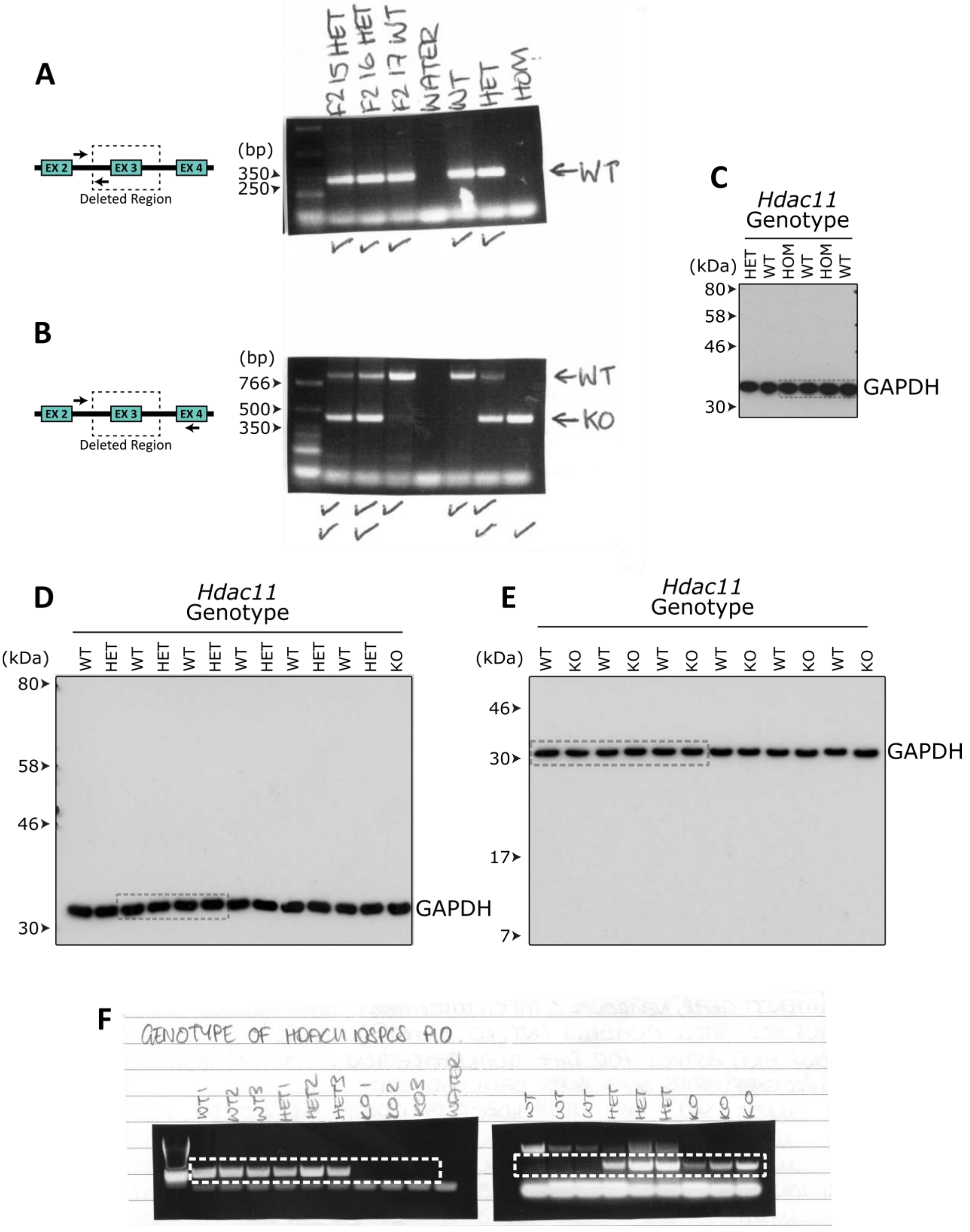

Supplementary Figure 8: Whole blots for reference proteins and DNA gels displayed in Fig. 4 and Fig. 6. A,B) Examples of uncropped DNA gels used for genotyping mice from the HDAC11 knockout line with the two different primer pairs. The samples in in panel B are those listed above panel A. **C-E)** Whole western blots for reference proteins displayed in Fig. 4. **F)** Uncropped DNA gels for genotypes shown in Fig. 6A. Dotted boxes indicate location of cropped images.

Supplementary Figure 9

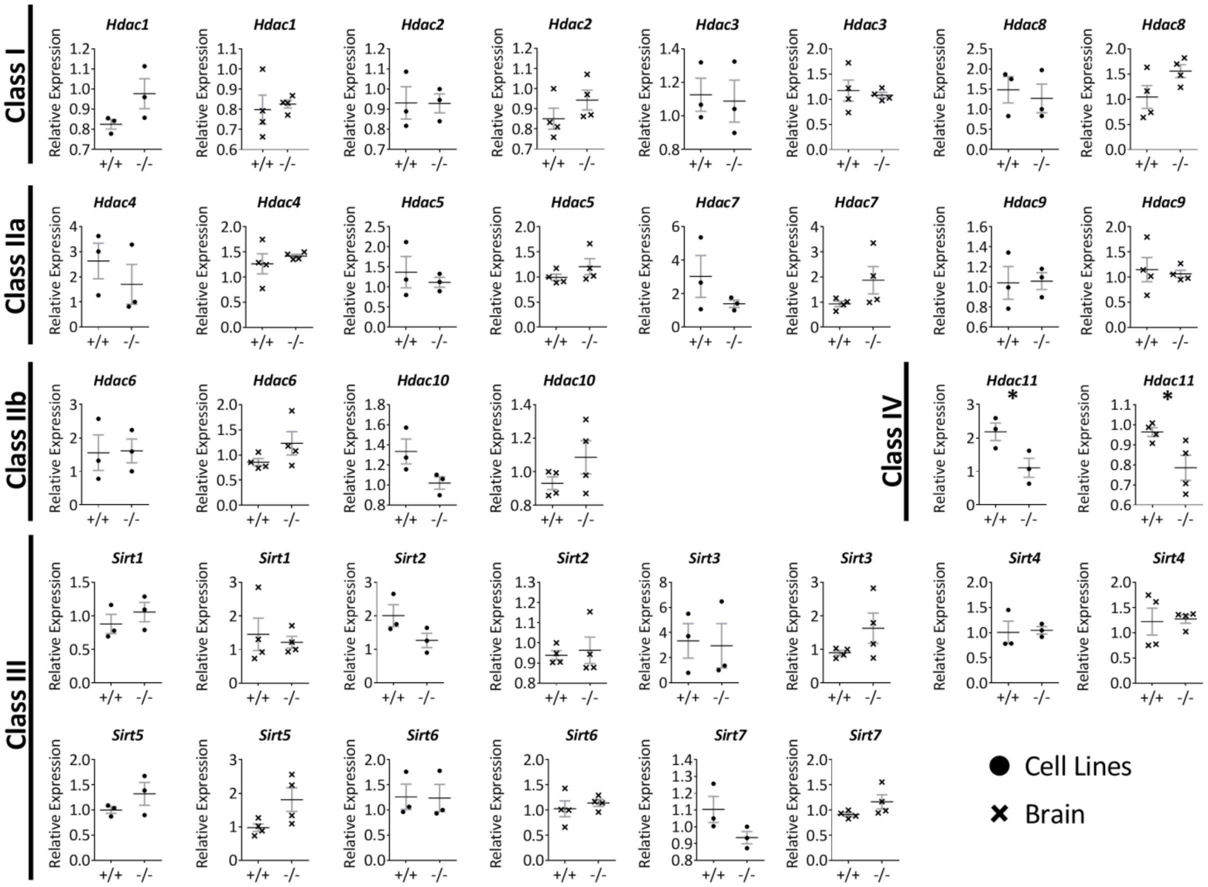

Supplementary Figure 9: Relative expression of all *Hdacs* in *Hdac11*$^{WT/WT}$ and *Hdac11*$^{KO/KO}$ tissue. Proliferating neural cell lines derived from *Hdac11*$^{KO/KO}$ mice were induced to differentiate for 3 days *in vitro*. The expression of all *Hdac* genes was examined in these cell lines (circles) and total brain lysates of *Hdac11*$^{KO/KO}$ mice (crosses). Note that the assay (Applied Biosystems, Catalog #Mm00523422_m1) used in this experiment targets the non-deleted region of the *Hdac11* transcript (exon boundary 5-6) and detects a transcript in *Hdac11*$^{KO/KO}$ neural cells / mouse brain, albeit at lower levels than *Hdac11*$^{WT/WT}$ tissue and predicted to result from nonsense mediated decay. N = 3 (cell lines), N = 4 (brains), circles/crosses = individual sample values, bars = mean ± SE, *p<0.05, two-tailed unpaired Student's t-test. Gene expression was normalised to the geomean of *Gapdh*, *Ubc* and *Atp5b*.

Supplementary Figure 10

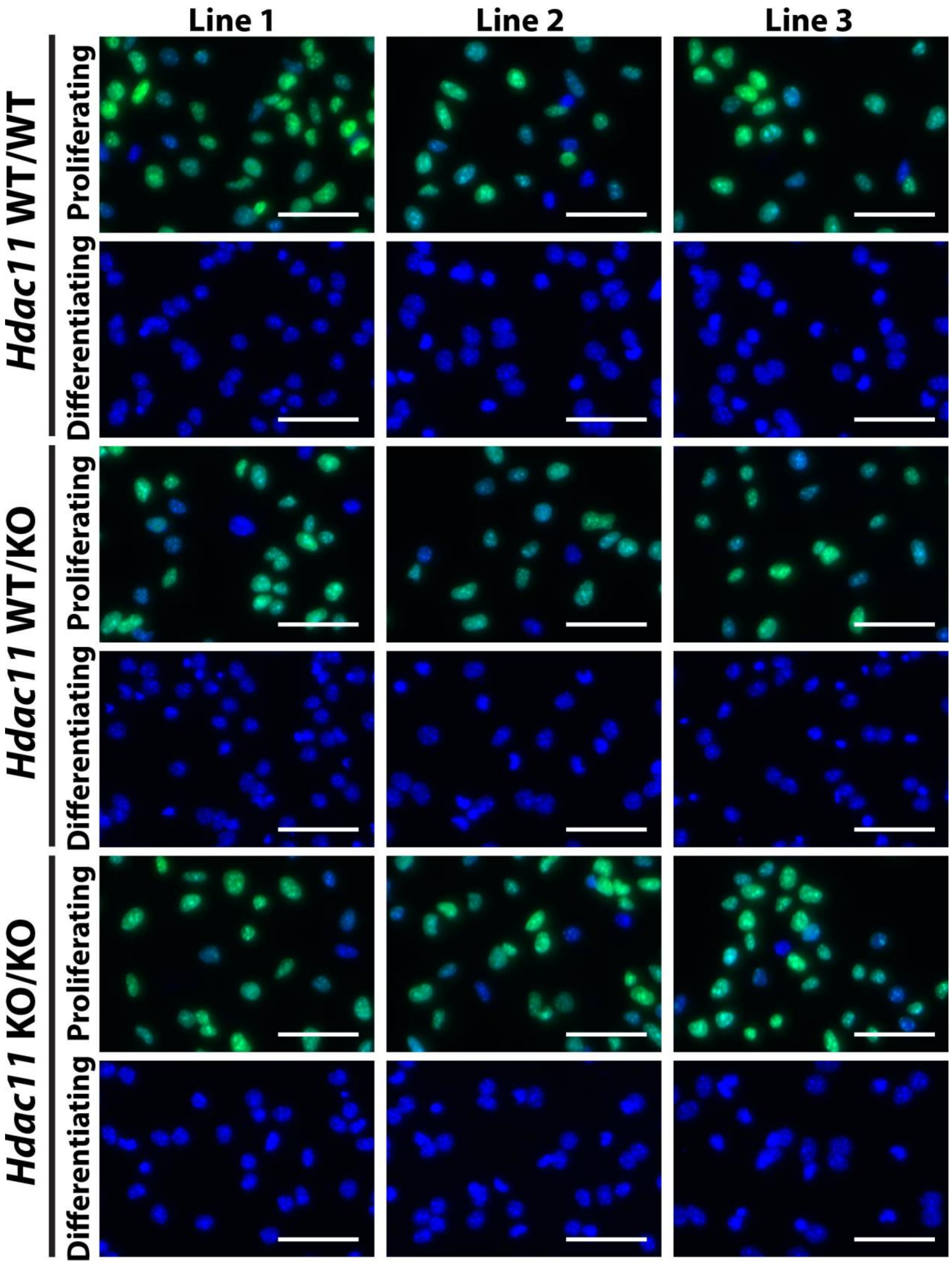

Supplementary Figure 10: Integration of EdU into proliferating neural cells derived from *Hdac11*$^{KO/KO}$ mice. Three neural cells lines were generated from E14.5 mice of each genotype (*Hdac11*$^{WT/WT}$, *Hdac11*$^{WT/KO}$ and *Hdac11*$^{KO/KO}$) and maintained in proliferation and differentiation inducing conditions for 3 days. On days 2-3 the cells were treated with EdU. Proliferating neural cells generated from mice of all genotypes display incorporation of EdU into the majority of cells. EdU was not incorporated into neural cells generated from all genotypes when they were maintained in differentiation inducing conditions. Scale bar = 50 µm.

Supplementary Figure 11

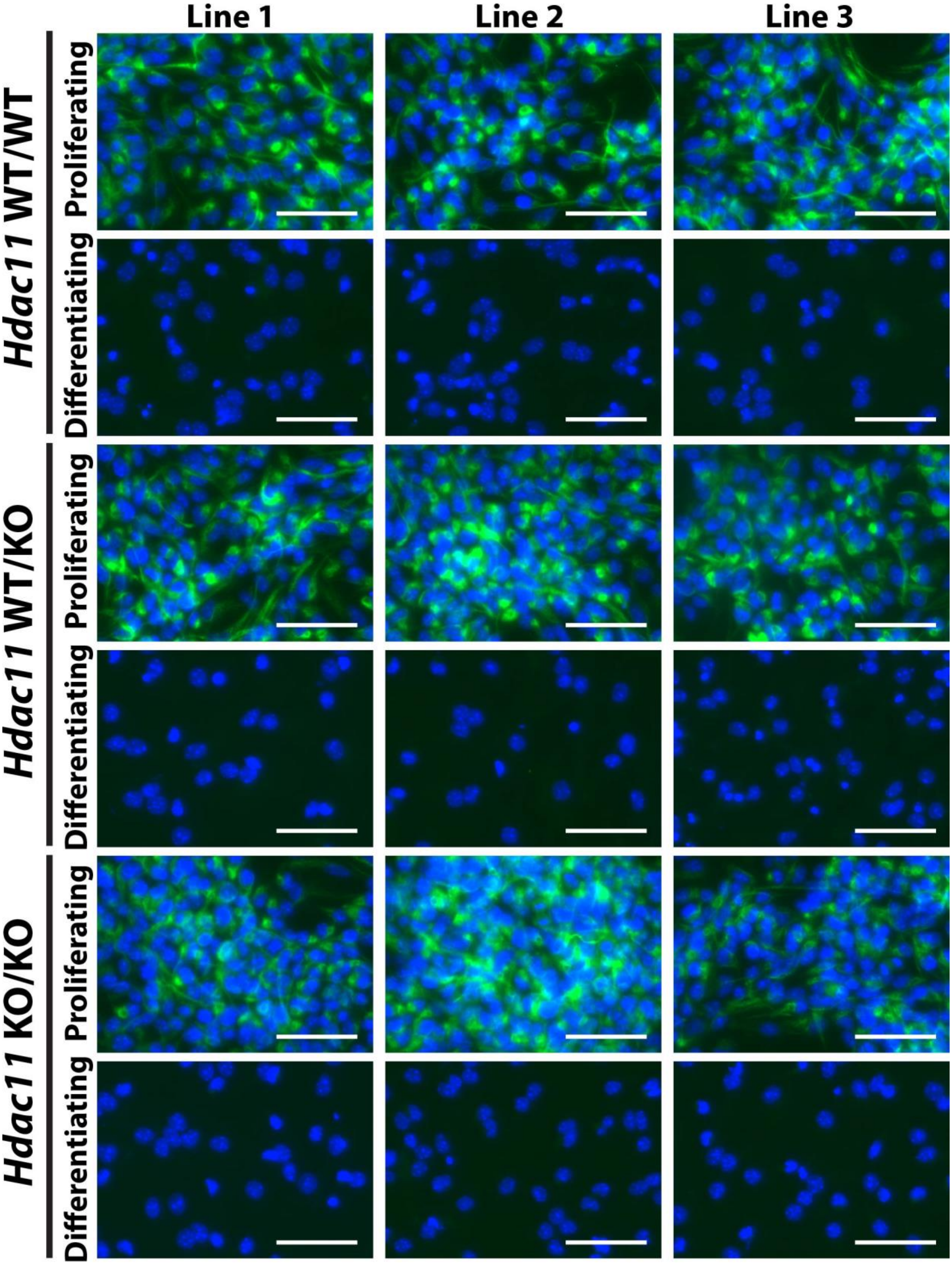

Supplementary Figure 11: Nestin expression in proliferating neural cells derived from *Hdac11*$^{KO/KO}$ mice. Three neural cells lines were generated from E14.5 mice of each genotype (*Hdac11*$^{WT/WT}$, *Hdac11*$^{WT/KO}$ and *Hdac11*$^{KO/KO}$) and maintained in proliferation and differentiation inducing conditions for 3 days. The majority of the cells were positive for neural stem cell associated marker nestin when the cells were maintained in proliferative conditions. Nearly all cells were negative for nestin when the cells were maintained in differentiation inducing conditions. Scale bar = 50 µm.

Supplementary Figure 12

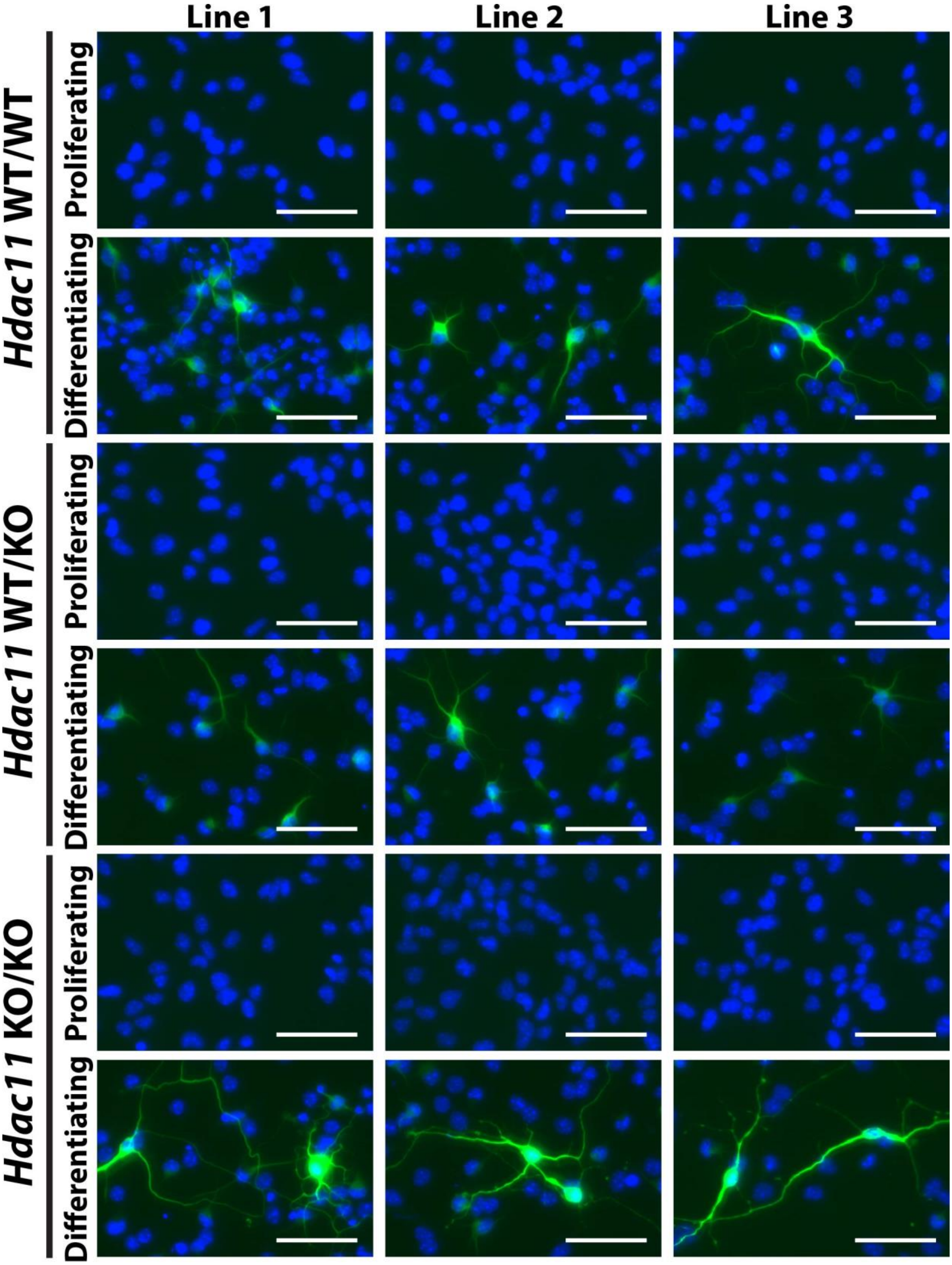

Supplementary Figure 12: TUBB3 expression in differentiating neural cells derived from *Hdac11*$^{KO/KO}$ mice. Three neural cells lines were generated from E14.5 mice of each genotype (*Hdac11*$^{WT/WT}$, *Hdac11*$^{WT/KO}$ and *Hdac11*$^{KO/KO}$) and maintained in proliferation and differentiation inducing conditions for 3 days. Nearly all cells were negative for neuron-associated marker TUBB3 when the cells were maintained in proliferative conditions. In contrast, cells positive for TUBB3 were readily identified when the cells were maintained in differentiation inducing conditions. Scale bar = 50 µm.

Supplementary Figure 13

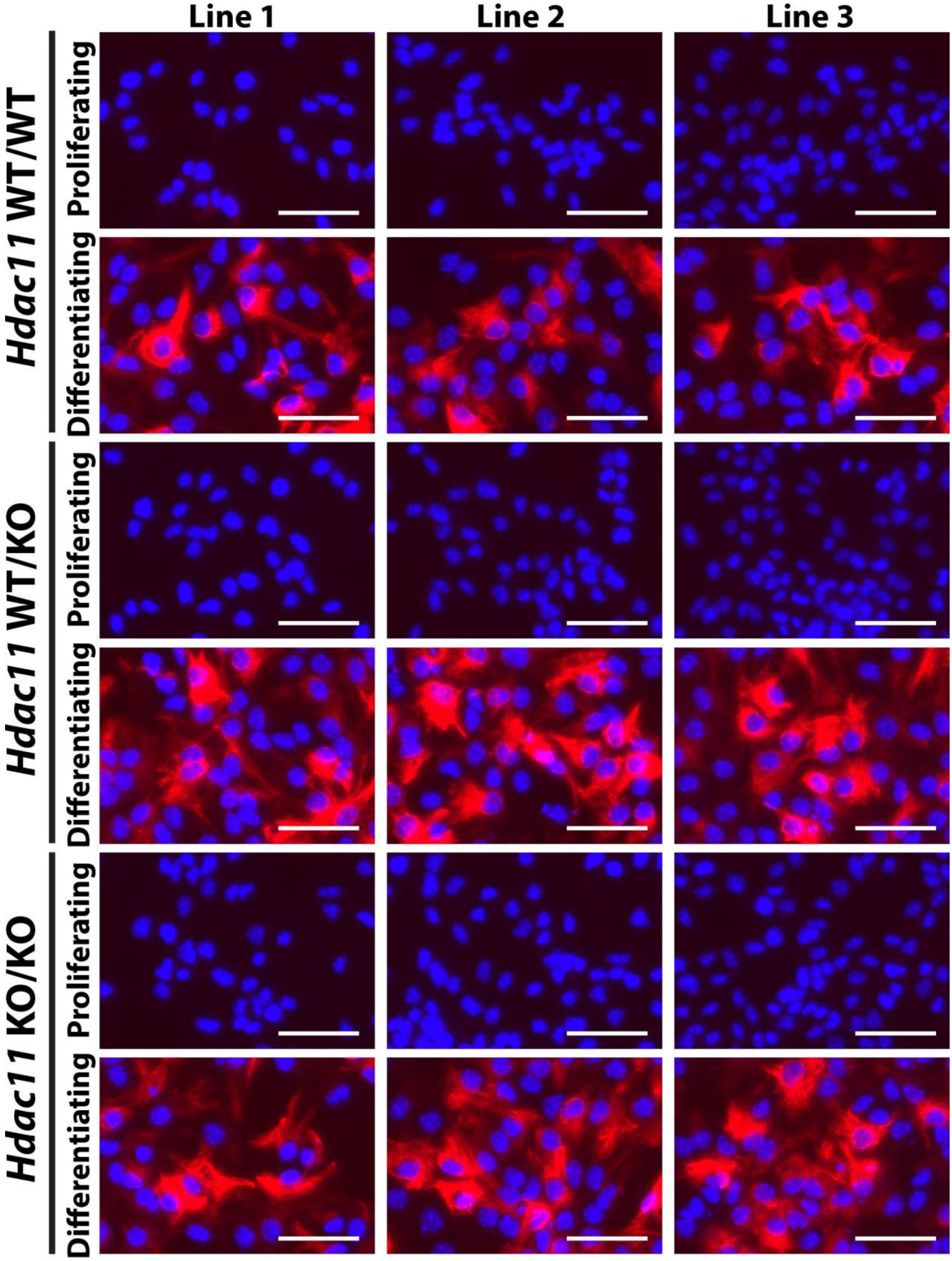

Supplementary Figure 13: GFAP expression in differentiating neural cells derived from *Hdac11*$^{\mathbf{KO/KO}}$ mice. Three neural cells lines were generated from E14.5 mice of each genotype (*Hdac11*$^{WT/WT}$, *Hdac11*$^{WT/KO}$ and *Hdac11*$^{KO/KO}$) and maintained in proliferation and differentiation inducing conditions for 3 days. The majority of the cells were negative for astrocyte associated marker GFAP when the cells were maintained in proliferative conditions. Whereas, many cells positive for GFAP are clearly identifiable when the cells were maintained in differentiation inducing conditions. Scale bar = 50 µm.

Supplementary Figure 14

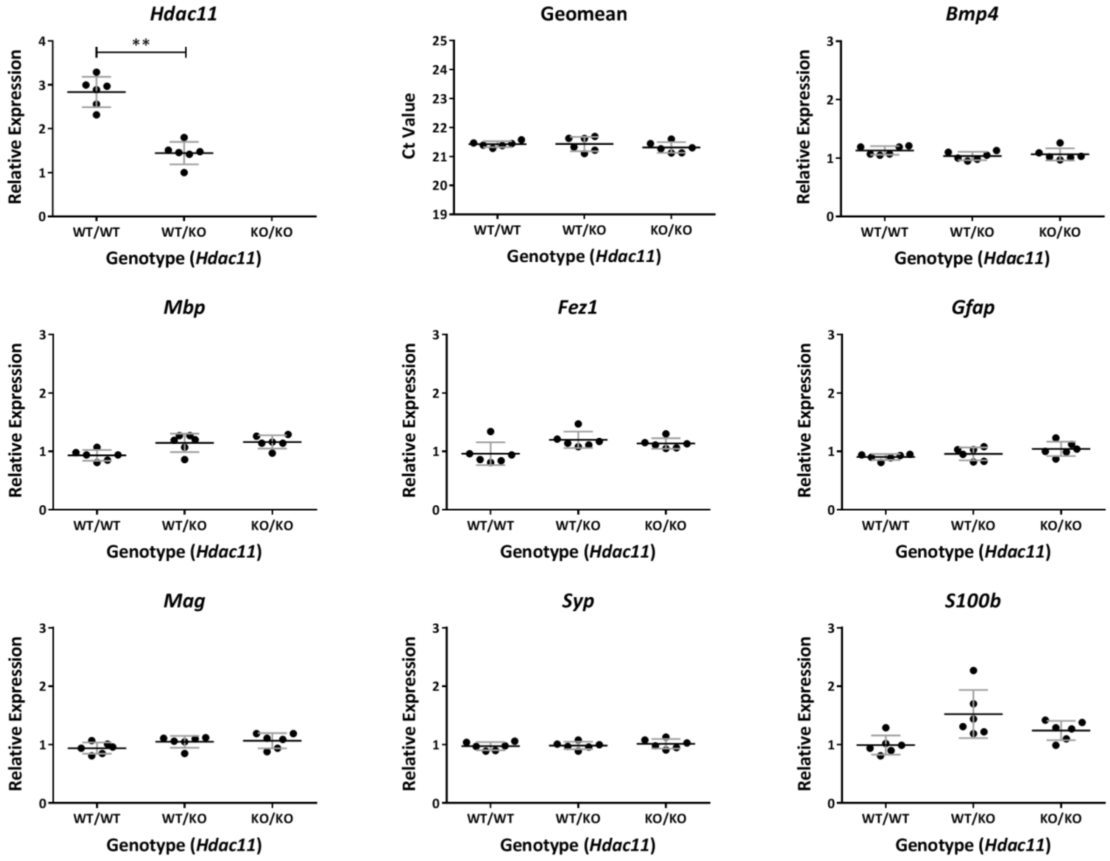

Supplementary Figure 14: Relative gene expression in total brain lysates of *Hdac11*$^{KO/KO}$ mice. There was no detectable impact on the expression of genes of interest in total brain lysates of *Hdac11*$^{KO/KO}$ mice. $N = 6$, dots = individual sample values, bars = mean ± SD, $^{**}p<0.01$, two-tailed unpaired Student's *t*-test. Gene expression was normalised to the mean of *Gapdh* and *Sdha*.

Supplementary Figure 15

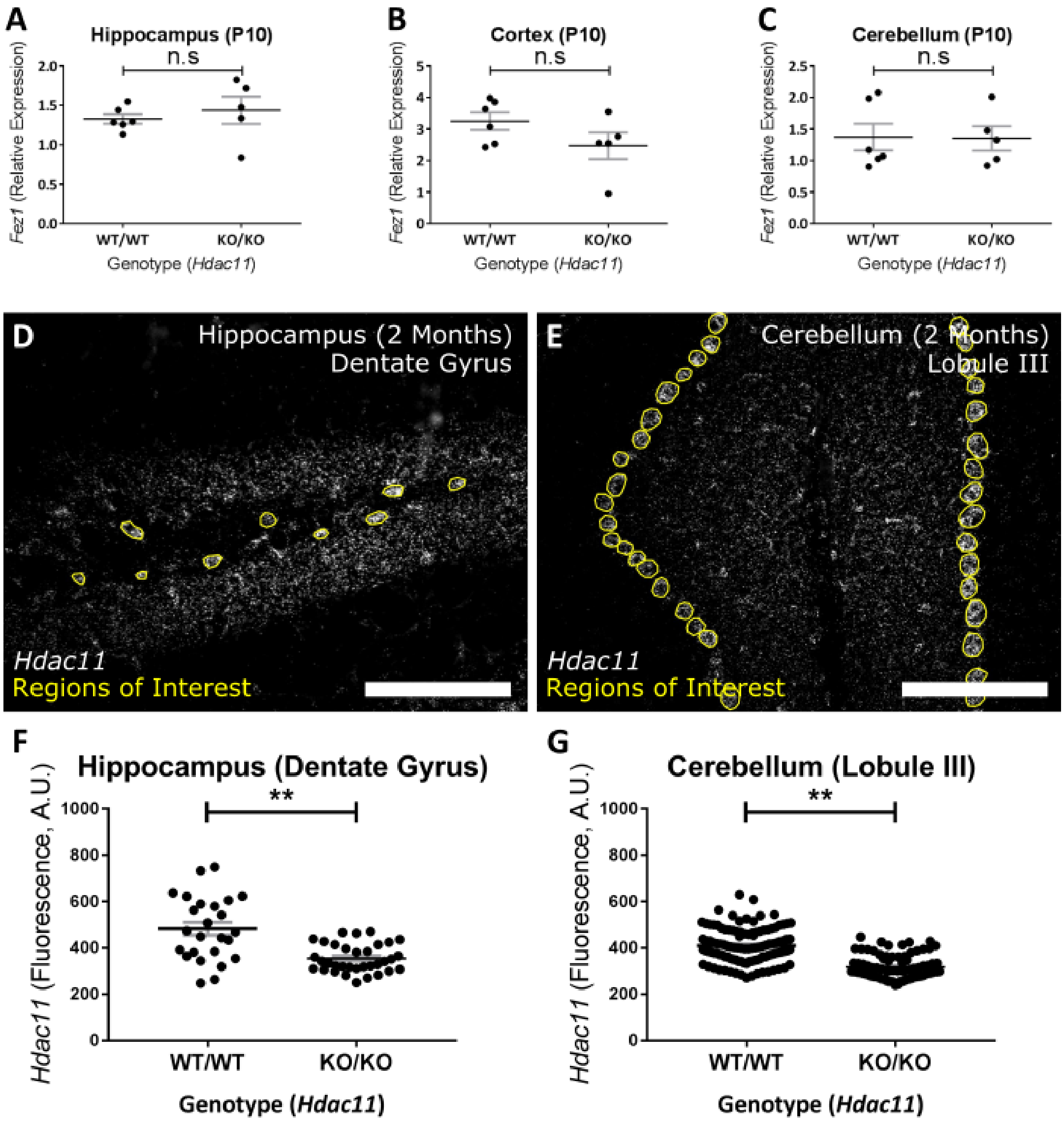

Supplementary Figure 15: Analysis brain region specific gene expression in *Hdac11*$^{KO/KO}$ adult mice using qPCR and *in situ* hybridisation. At P10, the **A)** hippocampus, **B)** cortex nor **C)** cerebellum displayed affected levels of *Fez1* expression in *Hdac11*$^{KO/KO}$ mice as measured by qPCR. Gene expression was normalised to the mean of *Canx* and *Sdha*. Images from *in situ* hybridisation (RNA scope) demonstrating how single *Hdac11* positive cells within the 2-month-old mouse **D)** dentate gyrus and **E)** cerebellum were selected (Regions of interest) for analysis of *Fez1* intensity. *Hdac11* expression was lower in the **F)** dentate gyrus and the **G)** cerebellum of *Hdac11*$^{KO/KO}$ mice. Scale bar = 200µm **(D-E)**. *N*=3(*n*≥25) (dots = individual sample values), bars = mean ± SE, **p<0.01, two-tailed unpaired Student's *t*-test **(F-G)**.

Supplementary Table 1

Antibody	Dilution	Time
**Rabbit anti-Ki67** (Genetex, Catalog #GTX16667)	1:250	2 Hrs
**Mouse anti-nestin** (BD Bioscience, Catalog #556309)	1:500	2 Hrs
**Mouse anti-TUBB3** (Promega, Catalog #G712A)	1:1500	2 Hrs
**Rabbit anti-GFAP** (DAKO, Catalog #Z0334)	1:500	2 Hrs
**Rabbit anti-HDAC11** (Sigma, Catalog #H4539)	1:200	15 Hrs
**Rabbit anti-HDAC11** (Abcam, Catalog #AB18973)	1:200	15 Hrs
**Rabbit anti-HDAC11** (Abcam, Catalog #AB135491)	1:200	15 Hrs
**Donkey anti-mouse IgG (H+L) AF488** (Life Technologies, Catalog #A21202)	1:500	1 Hr
**Donkey anti-rabbit IgG (H+L) AF488** (Life Technologies, Catalog #A21206)	1:500	1 Hr
**Donkey anti-rabbit IgG (H+L) AF594** (Life Technologies, Catalog #A21207)	1:500	1 Hr

Supplementary Table 1: Antibody incubation details for immunocytochemistry. Primary antibodies were diluted in DPBS made up of 0.1% Triton X-100 and 10% FBS. Secondary antibodies were diluted in DPBS made up of 0.1% Triton X-100 and 5% FBS. ≤2 hour incubations were carried out at room temperature whereas 15 hour incubations were kept at 4°C.

Supplementary Table 2

Gene	Assay	Slope	R2	Efficiency	*E* ($10^{(-1/slope)}$)
Hdac1	Mm02391771_g1	3.2185	0.9956	104.30	2.04
Hdac2	Mm00515108_m1	3.2835	0.9987	101.58	1.92
Hdac3	Mm00515916_m1	3.5171	0.9972	92.38	1.92
Hdac4	Mm01299543_m1	3.4047	0.9995	96.64	1.97
Hdac5	Mm01246076_m1	3.1893	0.9959	105.65	2.06
Hdac6	Mm01341125_m1	3.2493	0.9974	103.01	2.03
Hdac7	Mm00469527_m1	3.4438	1.0000	95.15	1.95
Hdac8	Mm01224977_m1	3.2937	0.9982	101.12	2.01
Hdac9	Mm01293999_m1	3.2618	0.9967	102.43	2.03
Hdac10	Mm01308118_g1	3.3169	0.9992	100.18	2.00
Hdac11	Mm01183513_m1	3.2922	0.9998	101.25	2.01
Sirt1	Mm00490758_m1	3.3817	0.9998	97.56	1.94
Sirt2	Mm01149204_m1	3.5177	0.9990	92.41	1.92
Sirt3	Mm00452129_m1	3.1664	0.9977	106.81	2.07
Sirt4	Mm01201915_m1	3.1388	0.9962	108.05	2.08
Sirt5	Mm00663721_m1	3.2027	0.9979	105.13	2.05
Sirt6	Mm01149042_m1	3.4577	0.9999	94.63	1.95
Sirt7	Mm00461895_m1	3.4861	0.9998	93.57	1.94
Actb	Mm00607939_s1	3.5435	0.9999	91.52	1.91
Gapdh	Mm99999915_g1	3.4969	0.9994	93.18	1.93

Supplementary Table 2: TaqMan® gene expression assays used to detect expression of *Hdac* genes. Serial dilutions (each 10-fold lower than the last) of cDNA were prepared to generate a standard curve for each TaqMan® assay. Using the slope of the curve, the primer efficiency (*E*) calculations were calculated according to the equation $\boldsymbol{E = 10^{[-1/slope]}}$ as described by Pfaffl[53]. *Actb* and *Gapdh* are included in this list because they were used for the comparative gene expression calculation described in Supplementary Table 6.

Supplementary Table 3

Assay	Gene Name
18S-Hs99999901_s1	Eukaryotic 18S rRNA
Msi2-Mm00475180_m1	Musashi homolog 2 (Drosophila)
Hes1-Mm00468601_m1	hairy and enhancer of split 1 (Drosophila)
Vcan-Mm01283063_m1	versican
Gnl3-Mm00463571_m1	guanine nucleotide binding protein-like 3 (nucleolar)
Ascl1-Mm03058063_m1	achaete-scute complex homolog 1 (Drosophila)
Aes-Mm00507847_m1	amino-terminal enhancer of split
Dlx2-Mm00438427_m1	distal-less homeobox 2
Mtap2-Mm00485230_m1	microtubule-associated protein 2
Artn-Mm00507845_m1	artemin
Tnr-Mm00659075_m1	tenascin R
Fez1-Mm00805945_m1	fasciculation and elongation protein zeta 1 (zygin I)
Syp-Mm00436850_m1	synaptophysin
Apoe-Mm00437573_m1	apolipoprotein E
S100b-Mm00485897_m1	S100 protein, beta polypeptide, neural
Gfap-Mm00546086_m1	glial fibrillary acidic protein
Slc1a3-Mm00600697_m1	solute carrier family 1 (glial high affinity glutamate transporter), member 3
Pdgfra-Mm00440701_m1	platelet derived growth factor receptor, alpha polypeptide
Galc-Mm00484646_m1	galactosylceramidase
Olig2-Mm01210556_m1	oligodendrocyte transcription factor 2
Canx-Mm00500330_m1	calnexin
Sdha-Mm01352366_m1	succinate dehydrogenase complex, subunit A, flavoprotein (Fp)
Cyc1-Mm00470540_m1	cytochrome c-1
Fabp7-Mm00445225_m1	fatty acid binding protein 7, brain

Supplementary Table 3: List of TaqMan® assays and abbreviations used in heat map on Figure 1. Genes were selected because they were reported or predicted to be differentially expressed in proliferating and differentiating neural cells *in vitro*.

Supplementary Table 4

Assay	Gene Name
18S-Hs99999901_s1	Eukaryotic 18S rRNA
Gapdh-Mm99999915_g1	glyceraldehyde-3-phosphate dehydrogenase
Actb-Mm00607939_s1	actin, beta
Sdha-Mm01352366_m1	succinate dehydrogenase complex, subunit A, flavoprotein (Fp)
Ubc-Mm02525934_g1	ubiquitin C
Atp5b-Mm00443967_g1	ATP synthase, H+ transporting mitochondrial F1 complex, beta subunit
Ntrk1-Mm01219406_m1	neurotrophic tyrosine kinase, receptor, type 1
Ntrk2-Mm00435422_m1	neurotrophic tyrosine kinase, receptor, type 2
Ntrk3-Mm00456222_m1	neurotrophic tyrosine kinase, receptor, type 3
Tac1-Mm01166996_m1	tachykinin 1
P2rx3-Mm00523699_m1	purinergic receptor P2X, ligand-gated ion channel, 3
Ret-Mm00436304_m1	ret proto-oncogene
Scn9a-Mm00450762_s1	sodium channel, voltage-gated, type IX, alpha
Scn10a-Mm00501467_m1	sodium channel, voltage-gated, type X, alpha
Trpv1-Mm01246302_m1	transient receptor potential cation channel, subfamily V, member 1
Pou4f1-Mm02343791_m1	POU domain, class 4, transcription factor 1
Phox2b-Mm00435872_m1	paired-like homeobox 2b
Neurog1-Mm00440466_s1	neurogenin 1
Neurog2-Mm00437603_g1	neurogenin 2
Runx1-Mm01213404_m1	runt related transcription factor 1
Runx3-Mm00490666_m1	runt related transcription factor 3
Pax6-Mm00443081_m1	paired box gene 6
Pou5f1-Mm03053917_g1	POU domain, class 5, transcription factor 1
Ascl1-Mm03058063_m1	achaete-scute complex homolog 1 (Drosophila)
Nanog-Mm02384862_g1	Nanog homeobox
Sox17-Mm00488363_m1	SRY-box containing gene 17
T-Mm01318252_m1	brachyury
Pax7-Mm01354484_m1	paired box gene 7
Sox10-Mm01300162_m1	SRY-box containing gene 10
Sox1-Mm00486299_s1	SRY-box containing gene 1
Foxg1-Mm02059886_s1	forkhead box G1
Syp-Mm00436850_m1	synaptophysin
Dlg4-Mm00492193_m1	discs, large homolog 4 (Drosophila)
Emx1-Mm01182609_m1	empty spiracles homeobox 1
Nkx2-1-Mm00447558_m1	NK2 homeobox 1
Slc17a7-Mm00812886_m1	solute carrier family 17 (sodium-dependent inorganic

	phosphate cotransporter), member 7
Slc17a6-Mm00499876_m1	solute carrier family 17 (sodium-dependent inorganic phosphate cotransporter), member 6
Dlk1-Mm00494477_m1	delta-like 1 homolog (Drosophila)
Isl1-Mm00517585_m1	ISL1 transcription factor, LIM/homeodomain
Prph-Mm00449704_m1	peripherin
Snai2-Mm00441531_m1	snail homolog 2 (Drosophila)
Snai1-Mm00441533_g1	snail homolog 1 (Drosophila)
Emx2-Mm00550241_m1	empty spiracles homeobox 2
En1-Mm00438709_m1	engrailed 1
Gbx2-Mm00494578_m1	gastrulation brain homeobox 2
Hoxb4-Mm00657964_m1	homeobox B4
Hoxb9-Mm01700220_m1	homeobox B9
Gad2-Mm00484623_m1	glutamic acid decarboxylase 2

Supplementary Table 4: List of TaqMan® assays and abbreviations used in heat map on Figure 3. Genes were selected because they were reported or predicted to be differentially expressed between the central and peripheral nervous system. Particularly with a specific intention to include genes expressed in the dorsal root ganglion.

Supplementary Table 5

Antibody	Temp.	Milk	Dilution	Time
Rabbit anti-HDAC11 **(Sigma, Catalog #H4539)**	4°C	1%	1:1000	15 Hrs
Rabbit anti-HDAC11 **(Abcam, Catalog #AB18973)**	4°C	1%	1:500	15 Hrs
Rabbit anti-HDAC11 **(Abcam, Catalog #AB135491)**	4°C	1%	1:500	15 Hrs
Rabbit anti-HDAC11 **(Millipore, Catalog #09-827)**	4°C	1%	1:500	15 Hrs
Mouse anti-synaptophysin (SYP) [SY38] **(Abcam, Catalog #8049)**	4°C	1%	1:5000	15 Hrs
Mouse anti-SNAP25 **(DB Transduction Laboratories, Catalog #610366)**	4°C	1%	1:5000	15 Hrs
Rabbit anti-mitofuscin 2 **(Abcam, Catalog #ab50838)**	4°C	1%	1.0 µg/ml	15 Hrs
Mouse anti-alpha tubulin, DM1A **(Sigma, Catalog #T9026)**	RT	None	1:40000	1 Hr
Rabbit anti-histone H3, CT, Pan **(Millipore, Catalog #07-690)**	RT	None	1:25000	1 Hr
Mouse anti-GAPDH **(Millipore, Catalog #MAB374)**	RT	None	1:2000	1 Hr
Goat anti-mouse IgG HRP **(Thermo Sci. Catalog #31430)**	RT	None	1:4000	1 Hr
Goat anti-rabbit IgG HRP **(DAKO, Catalog #P0448)**	RT	None	1:4000	1 Hr
Rabbit anti-goat IgG HRP **(DAKO, Catalog #PO449)**	RT	None	1:4000	1 Hr

Supplementary Table 5: Antibody incubation details for western blotting. Reagents were diluted in PBS made up with 0.1% Tween-20. Blots were incubated on a shaker to maintain movement of the solution.

Supplementary Table 6

Gene	Expression	± StdDev
Actb	23.52	1.84
Sirt2	5.78	0.60
Hdac2	2.62	0.21
Hdac11	**0.98**	**0.11**
Hdac3	0.80	0.20
Hdac5	0.62	0.11
Hdac9	0.55	0.15
Sirt1	0.53	0.40
Hdac1	0.53	0.03
Hdac4	0.33	0.08
Hdac6	0.23	0.01
Sirt3	0.23	0.03
Sirt7	0.22	0.02
Hdac10	0.15	0.02
Sirt6	0.13	0.04
Hdac8	0.12	0.04
Sirt5	0.10	0.02
Sirt4	0.06	0.03
Hdac7	0.05	0.01

Supplementary Table 6: Comparative expression of *Hdac* genes and *Actb* in mouse brain. Samples were cDNA prepared from lysates of whole brain tissue from *Hdac11*$^{WT/WT}$ mice (*N*=4). Expression between genes was calculated according to method reported by Pfaffl (2001) using the formula $\boldsymbol{E}^{-(\mathbf{Ct\ Target} - \mathbf{Ct\ Ref})}$. In this formula: *E* = primer efficiency; Ct Target = crossing threshold of gene of interest; and Ct Ref = crossing threshold of *Gapdh*. Primer efficiencies are listed on Supplementary Table 2. This calculation was carried out for each sample. The value of each sample was then normalised to the median value of that calculated for *Hdac11*. The average expression value and standard deviation are listed in the table.

Supplementary Table 7

Gene	Proliferating	Differentiating
Msi2	0.1043	0.2404
Hes1	0.3719	0.8615
Vcan	0.6496	0.4552
Gnl3	0.4820	0.5971
Ascl1	0.9146	0.0655
Aes	0.0773	0.0027
Dlx2	0.3434	0.0662
Mtap2	0.0314	0.0576
Artn	0.3574	0.1640
Tnr	0.8898	0.0008
Fez1	0.0092	0.0296
Syp	0.9329	0.8043
Apoe	0.5693	0.1843
S100b	0.8535	0.0466
Gfap	0.0098	0.0601
Slc1a3	0.2866	0.5925
Pdgfra	0.1909	0.0982
Galc	0.5866	0.8341
Olig2	0.7073	0.7949
Cyc1	0.1178	0.6539
Fabp7	0.2811	0.0031

Supplementary Table 7: List of *p*-values from analysis of gene expression profile in neural cells derived from *Hdac11*$^{WT/WT}$ and *Hdac11*$^{KO/KO}$ mice. *p*-values were calculated from comparing the 2^-ΔΔCt of neural cells derived from *Hdac11*$^{WT/WT}$ and *Hdac11*$^{KO/KO}$ mice in a) proliferative conditions and b) differentiation inducing conditions. Statistically significant values are highlighted. The 2^-ΔΔCt values used in this analysis correspond to those illustrated in heat map shown in Fig. 6E and the *p*-values were calculated using a two-tailed t-test, *N*=3, shaded boxes = $p<0.05$.

Supplementary Table 8

	Hippocampus		Cortex		Cerebellum		Spinal C.
**Gene**	**2 Mth**	**P10**	**2 Mth**	**P10**	**2 Mth**	**P10**	**2 Mth**
Hdac11	0.006	<0.001	<0.001	<0.001	<0.001	0.002	0.034
Fez1	0.005	0.526	0.822	0.146	0.627	0.943	0.950
Mbp	0.107	0.879	0.781	0.074	0.849	0.635	0.361
Plp1	0.145	0.727	0.633	0.086	0.467	0.954	0.331

Supplementary Table 8: List of *p*-values from analysis of *Hdac11*, *Fez1*, *Mbp* and *Plp1* gene expression in neural tissue dissected from *Hdac11*$^{\mathbf{WT/WT}}$ and *Hdac11*$^{\mathbf{KO/KO}}$ mice. *p*-values were calculated from comparing the 2^-ΔΔCt of tissue dissected from *Hdac11*$^{WT/WT}$ and *Hdac11*$^{KO/KO}$ mice. Spinal cord tissue was only dissected from 2-month-old mice. Statistically significant values are highlighted. *N*=5-6, two-tailed unpaired Student's *t*-test, shaded boxes = $p<0.05$.
